# What Machine Learning Can Tell Us About the Role of Language Dominance in the Diagnostic Accuracy of German LITMUS Non-word and Sentence Repetition Tasks

**DOI:** 10.3389/fpsyg.2018.02757

**Published:** 2019-01-30

**Authors:** Lina Abed Ibrahim, István Fekete

**Affiliations:** ^1^Department of English, University of Oldenburg, Oldenburg, Germany; ^2^Department of Dutch, University of Oldenburg, Oldenburg, Germany

**Keywords:** bilingualism, specific language impairment, sentence repetition, non-word repetition, language dominance, k-medoid clustering algorithm, unsupervised learning, conditional inference trees

## Abstract

The present study investigates the performance of 21 monolingual and 56 bilingual children aged 5;6–9;0 on German LITMUS-sentence-repetition (SRT; Hamann et al., 2013) and non-word-repetition-tasks (NWRT; Grimm et al., 2014), which were constructed in accordance with the **LITMUS**-principles (**L**anguage **I**mpairment **T**esting in **M**ultilingual **S**ettings; Armon-Lotem et al., 2015). Both tasks incorporate phonologically and syntactically complex structures shown to be cross-linguistically challenging for children with Specific Language Impairment (SLI) and aim at minimizing bias against bilingual children while still being indicative of the presence of language impairment across language combinations (see Marinis and Armon-Lotem, 2015; for sentence-repetition; Chiat, 2015 for non-word-repetition). Given the great variability in bilingual language exposure and the potential effect of language experience on language performance in bilingual children, we examined whether background variables related to bilingualism, particularly, the degree language dominance as measured by relative amount of use and exposure, could compromise the diagnostic accuracy of the German LITMUS-SRT and NWRT. We further investigated whether a combination of the two tasks provides better diagnostic accuracy and helps avoid cases of misdiagnosis. To address this, we used an unsupervised machine learning algorithm, the Partitioning-Around-Medoids (PAM, Kaufman and Rousseeuw, 2009), for deriving a clinical category for the children as ± language-impaired based on their performance scores on SRT and NWRT (in isolation and combined) while withholding information about their clinical status based on standardized assessment in their first (home language, L1) and second language (societal language, L2). Subsequently, we calculated diagnostic accuracy and used regression analysis to investigate which background variables (age of onset, length of exposure, degree of language dominance, socio-economic-status, and risk factors for SLI) best explained clinical-group-membership yielded from the PAM-analysis based on the children’s NWRT and SRT performance scores. Results show that although language-dominance clearly influences the performance of bilingual typically developing children, especially in the SRT, the diagnostic accuracy of the tools is not compromised by language dominance: while risk factors for SLI were significant predictors for clinical group membership in all models, language dominance did not contribute at all to explaining clinical cluster membership as typically developing or SLI based on any of the combinations of the SRT and NWRT variables. Additionally, results confirm that a combination of SRT scored by correct target structure and the structurally more complex language-dependent part of the NWRT yields better diagnostic accuracy than single measures and is only sensitive to risk factors for SLI and not to dominance levels or SES.

## Introduction

Recent research in language disorders has focused on problems of language assessment and the identification of what is currently referred to in the literature as Developmental Language Disorder (DLD, see Bishop et al., 2017) or Specific Language Impairment (SLI^1^) in bilingual children. The latter term refers to a disorder in the development of language in the absence of auditory, cognitive, sensory-motor, neurological, or socio-emotional deficits (Leonard, 1998, 2014). A challenge constantly facing clinicians is to determine whether a bilingual child’s poor performance on language tasks in the societal language (second language-L2) is due to an inborn language impairment (LI) or to insufficient exposure to the L2 (cf. Armon-Lotem et al., 2015; Marinis et al., 2017).

A major contributor to the diagnostic difficulties of SLI is the heterogeneity of children with SLI, who constitute a group with diverse linguistic profiles and deficits of varying severity across language components (Crutchley et al., 1997; Conti-Ramsden et al., 2001; Friedmann and Novogrodsky, 2011; Leonard, 2014 among others). For many children with SLI, deficits in the area of morphosyntax (grammatical morphology and syntactic structure) stand out (Leonard, 2007; Marinis and van der Lely, 2007; Marinis, 2011). On the one hand, certain complex syntactic structures with linguistic operations involving dependencies such as syntactic movement (e.g., Wh-questions) and embedding (e.g., relative clauses), have been shown to be cross-linguistically problematic for children with SLI (Jakubowicz et al., 1998; van der Lely, 1998; Friedmann and Novogrodsky, 2011; Jakubowicz, 2011; Hamann and Tuller, 2014; Hamann et al., 2017). On the other hand, SLI may manifest itself differently depending on the language being acquired so that clinical markers vary across languages.

Problems of language-impaired children are not restricted to the morphosyntactic domain, albeit being most deficient. Various studies have shown that children with SLI also evince deficits in the area of phonology. These children lag behind their age matched peers in the acquisition of consonants and are particularly sensitive to phonological complexity such as consonant clusters (Gallon et al., 2007; Ferré et al., 2015; dos Santos and Ferré, 2018; Grimm and Hübner, in press), coda position (Tamburelli and Jones, 2013) and syllabic position in the foot (Bortolini and Leonard, 2000). As a coping strategy, consonant clusters are often reduced or even avoided (Bortolini and Leonard, 2000; Orsolini et al., 2001; Marshall et al., 2003). Although morphosyntactic and phonological deficits are more commonly reported in the literature (Leonard, 2014), children with SLI also have deficient lexical retrieval abilities, which are not only delayed but also qualitatively different from those of children with typical language development (Novogrodsky and Kreiser, 2015). A number of studies have further shown that children with SLI exhibit deficits in the interface between syntax-semantics and pragmatics, e.g., universal quantification, telicity and exhaustivity in Wh-questions (Roeper, 2004; Schulz and Roeper, 2011). Even though children with SLI often present different combinations of the deficits, Friedmann and Novogrodsky (2008, p. 214) point to the existence of “selective impairments in one module of language, and not in others.” Accordingly, it is possible “to identify subgroups within SLI with selective deficits in various language modules: syntax [grammatical/syntactic-SLI], lexicon [lexical-SLI], phonology [phonological-SLI] and pragmatics [pragmatic-SLI]” (ibid., p. 214).

Aside from the aforementioned language deficits, a large body of research has identified deficits in phonological short-term memory, as indicated by poor performance on repeating non-words with a length of two to four syllables as a special weakness in children with SLI (Gathercole and Baddeley, 1990; Archibald and Gathercole, 2006; Gathercole, 2006; for a meta-analysis see Graf Estes et al., 2007). Although deficits in phonological short-term memory and certain aspects of grammar involving grammatical computational aspects^2^ such as verbal morphology and syntactic comprehension often co-occur in children with SLI, evidence from a twin study by Bishop et al. (2006) has shown that despite being significantly heritable, the two vulnerable areas were separable. While some children displayed deficits in both areas, other children displayed deficits in one but not the other, suggesting that they are not “different manifestations of the same underlying deficit” (Leonard, 2014, p. 19).

Apart from diagnostic difficulties caused by the heterogeneity of the disorder, identifying LI in bilingual children is made far more complex by the great variability in their (typical) language development, which is influenced by a multitude of child internal and external factors (Paradis, 2011; Hamann, 2012). The latter include age of onset (AoO) of systematic (sustained) exposure to the second language (L2), length of exposure (LoE), quantity and quality of linguistic input (poor or enriched), L1-L2 typological proximity, status of the home language (high prestige, minority, or heritage language), and socioeconomic status (SES). The interplay of these factors makes it notoriously difficult to establish what is typical for bilingual language development (Tuller et al., 2018). Depending on the timing of exposure, bilingual children could be classified as simultaneous (AoO < 3), early (3 ≤ AoO < 4) or late (AoO ≥ 4) sequential child bilinguals (also referred to as child L2, Meisel, 2009). Even in simultaneous bilingual language acquisition, bilingual children “have their input space divided” (Paradis and Genesee, 1996, p. 9) and are likely to receive less exposure to each language, on average, than monolingual age peers acquiring the respective languages. As a result, bilingual children often develop unbalanced command of their two languages, i.e., their linguistic abilities are unevenly distributed both within and across language domains at a given age (e.g., Döpke, 2000; Yip and Matthews, 2006; Kohnert, 2010). The language with the more advanced state of development within the process of language acquisition (Deuchar and Muntz, 2003; Genesee and Nicoladis, 2007; Gathercole, 2016) or the language to which the child receives more exposure on a regular basis (Pearson et al., 1997) is commonly described as the dominant (stronger) language as opposed to the weaker or non-dominant one (see also Meisel, 2007). In this sense, dominance is associated with language exposure/use (Grosjean, 2016) and/or with the degree of proficiency in either language (Petersen, 1988; Deuchar and Muntz, 2003; Genesee and Nicoladis, 2007). In the present study, we adopt Argyri and Sorace’s (2007, p. 83) definition of dominance as “the language in which the bilingual child obtains more input on a regular basis” (see also Grosjean, 2010). Language dominance can also shift over time due to changes in patterns of use and exposure resulting from “changes in family structure, child-care arrangements, schooling, or place of residence” (Paradis, 2010: p. 652). For example, in case of early sequential child bilinguals, who start acquiring the societal (second language L2) while their home language (first language, L1) is still at an early developmental stage, a change in the degree of dominance is frequently observed with schooling (cf. Flores, 2015; de Houwer and Bornstein, 2016). Diagnostic problems particularly occur when bilingual children are solely assessed using monolingual norm-referenced tests in the majority/societal language, which might still be their weaker, i.e., non-dominant language at the time of assessment. In many cases, performance below monolingual average, especially on standardized measures for vocabulary and morphosyntax, is taken as evidence for LI leading to overdiagnosis with SLI (Bedore and Peña, 2008; Grimm and Schulz, 2014).

In addition to the aforementioned quantitative performance differences, a growing body of research has shown that the developmental trajectory of bilingual child language acquisition may show (persistent) delays (Tuller et al., 2015; Paradis et al., 2016) or temporary overlap with that of monolingual children with SLI (MoSLI), particularly in the area of morphosyntax (see Paradis, 2010 for an overview). The overlap in linguistic error patterns of bilingual typically developing children (BiTD) and error patterns serving as diagnostic markers for SLI in a particular language, e.g., extended use of infinitives in English (Rice and Wexler, 1996), object clitic omission in French (Paradis et al., 2003; Paradis, 2010; Hamann, 2012) and problems with SVA combined with the use of infinitives and verb placement errors in German (Clahsen, 1991; Hamann et al., 1998; Rothweiler et al., 2012) complicates the diagnosis of SLI in bilingual children. The delayed or deviant linguistic development of a bilingual child may be erroneously ascribed to bilingualism (underdiagnosis), while a child L2 learner may be overdiagnosed with SLI if such deficits are viewed as a token for SLI (Genesee et al., 2004; Grimm and Schulz, 2014; Armon-Lotem and de Jong, 2015), which could have costly consequences for the child and the society (Zurer-Pearson, 2010).

To avoid cases of misdiagnosis, it has been recommended to evaluate a bilingual child at least in her dominant language (Fredman, 2006) and ideally in both of her languages (American Speech-Language-Hearing Association [ASHA], 2004; Royal College of Speech and Language Therapists Specific Interest Group in Bilingualism [RCSLT], 2007; International Association of Logopedics and Phoniatrics [IALP], 2011), as genuine LI affects both. However, L1-assessment is often not feasible due to the lack of standardized language tests for (bilingual) children in their L1. Even if available, results may be unreliable due to incomplete L1-acquisition and/or L1-attrition, which are often reported for heritage language speakers (Montrul, 2008; Benmamoun et al., 2013). Not to mention that evaluation in two languages is time-consuming and that some of the immigrant L1 varieties undergo language change as a result of contact with the majority/societal language (L2), e.g., Immigrant Turkish in Germany (see Schroeder and Dollnick, 2013; Chilla and Şan, 2017). Hamann and Abed Ibrahim (2017) showed that even when dominance-adjusted bilingual cut-off criteria (Thordardottir, 2015) were applied to the standardized L1 tests, more than a quarter of the L1-dominant children in their sample were classified as SLI by the L1-tests. The fact that the latter children performed within aged-expectations on the L2-tests albeit being dominant in their heritage language questions the applicability of L1 tests in heritage contexts (even with norm adjustments) and suggests that direct assessment measures in the L2 are more reliable for identifying LI in bilingual populations, especially in case of heritage language speakers. This in turn makes it crucial to develop reliable tools that could disentangle effects of bilingualism and LI in bilingual contexts.

### The LITMUS Tools for Bilingual Language Assessment

In an attempt to cope with the diagnostic challenges in bilingual populations, a battery of tools was designed during COST Action IS0804 “Language Impairment in a Multilingual Society: Linguistic Patterns and the Road to Assessment” according to a set of linguistic principles that allow cross-linguistic comparability. These tools aim at minimizing the effect of factors related to bilingualism, so that SLI can be reliably identified in bilingual children with different language combinations. The latter tools are known as the LITMUS tools (Language Impairment Testing in Multilingual **S**ettings, see Armon-Lotem et al., 2015), among which are sentence repetition (SRTs) and non-word repetition tasks (NWRTs) and the Questionnaire for Parents of Bilingual Children (PaBiQ; Tuller, 2015). The latter was developed for gathering background information on factors related to bilingualism as well as information about risk factors for SLI. Such information is invaluable for the interpretation of performance results on linguistic tasks. In the current study, we concentrate on sentence repetition and non-word repetition (NWR) since they have been shown to reliably identify SLI in monolinguals (Conti-Ramsden et al., 2001) and to be less reliant on prior language experience than other language measures in bilinguals, e.g., receptive vocabulary (Chiat et al., 2013; Thordardottir and Brandeker, 2013). Depending on their construction, SRTs and NWRTs can be designed to not only assess (phonological) working memory (Archibald and Gathercole, 2006), but also the command of syntactic and phonological representations/derivations (see Polišenská et al., 2015 for sentence-repetition; Gallon et al., 2007 for non-word-repetition). Such linguistic representations/derivations, especially their complexity, have been shown to crucially influence performance in these tasks (e.g., Ferré et al., 2012; Friedmann et al., 2015) so that it has been argued that they are not mere measures of working memory (Vinther, 2002; Polišenská et al., 2015). Because of this versatility, they are ideal for targeting language-specific (LS) as well as cross-linguistically challenging syntactic/phonological structures while minimizing avoidance strategies (see Hamann et al., 2017 for SRT).

Sentence repetition taps morphosyntactic abilities as recalling a sentence involves processing of the incoming input string, analysis and reconstruction thereof, especially when the sentences are long enough to prevent mere phonological reiteration (Baddeley, 2000; Marinis and Armon-Lotem, 2015). Furthermore, compared to other types of tasks, it is less constrained by pragmatic and discourse factors (Polišenská et al., 2015; Hamann et al., 2017), and is thus often used in clinical assessment as a measure of sentence-level abilities. The German LITMUS-SRT (Hamann et al., 2013) under investigation here was constructed according to the LITMUS principles (Marinis and Armon-Lotem, 2015) and builds on the notion of linguistic computational complexity. Within the generative framework, computational complexity can be determined by the number and nature (e.g., merge vs. movement, distance of dependencies, and depth of embedding) of syntactic operations necessary for deriving a syntactic structure (Gibson, 1998; Jakubowicz, 2005; Hamann et al., 2007; Jakubowicz and Tuller, 2008; Friedmann et al., 2009). Children with atypical language acquisition are proposed to have a greater deficit on constructions with a higher degree of computational complexity, as the latter are more taxing to working memory capacities (Chomsky, 2005; Hamann et al., 2007; Jakubowicz and Tuller, 2008). A particular difficulty for children with SLI has been reported for structures involving movement along with intervening elements between the source of the moved constituent and its landing site, e.g., object Which-questions and object relative clauses with a lexical subject (Rizzi, 2004; Friedmann et al., 2015). Unlike the problems encountered by children with SLI, bilingual children with typical language development (BiTD) might struggle with vocabulary and uninterpretable features, i.e., grammatical features lacking semantic content like number agreement on the verb (Tsimpli and Dimitrakopoulou, 2007), or might even avoid complexity (Tuller et al., 2015). They are; however, assumed to have an intact language faculty and WM. Thus, having been acquired in the L1, syntactic operations such as recursion, embedding and movement do not have to be acquired again and should not be problematic for them given sufficient exposure to the L2 (Roeper, 2011). Accordingly, the German LITMUS-SRT incorporates a set of syntactically complex, i.e., computationally more demanding structures identified as difficult for children with SLI cross-linguistically in addition to a set of structures reported to be challenging for German MoSLI children such as topicalization and the sentence bracket, which represent crucial milestones in the acquisition of German word-order properties. The complex structures involve computational operations like syntactic movement (measured, for example by number of overt movement operations), in particular Wh-movement, i.e., fronting of interrogative or relative pronouns (Hamann et al., 1998; van der Lely, 1998; Marinis and van der Lely, 2007; Jakubowicz, 2011), and/or clausal embedding, e.g., relative clauses (Friedmann and Novogrodsky, 2011; Hamann and Tuller, 2014; Scheidenes and Tuller, 2018).

It has been recently shown that SRTs eliciting structures involving the latter operations can be reliably used to tease apart typically developing bilingual children from monolingual and bilingual children with SLI, not only in bilingual but also in bialectal settings (e.g., Armon-Lotem and Meir, 2016; Meir et al., 2016, 2017 for LITMUS-SRT in Russian and Hebrew; de Almeida et al., 2017; Fleckstein et al., 2018 for French; Lein et al., 2016; Abed Ibrahim and Hamann, 2017; Hamann et al., 2017; Hamann and Abed Ibrahim, 2017 for German; Theodorou et al. (2017) for Cypriot-Greek; see also Marinis et al., 2017 for an overview). In particular, Armon-Lotem and Meir (2016) showed that although the highest level of diagnostic accuracy can be achieved using a combination of SRTs in the child’s L1/Russian and L2/Hebrew (applying bilingual cut-offs), good diagnostic accuracy can still be achieved if SRT is only administered in the societal language (L2-Hebrew). In the same vein, Abed Ibrahim et al. (2018) and Chilla et al. (in press) looked into the potential influence of L1-L2 typological differences on the performance of bilingual children with Arabic, Portuguese, and Turkish as L1 on German LITMUS-SRT. L1-influence surfaced neither in the overall performance nor in the performance on the individual structures included in the task or in the expected L1-driven error patterns confirming the applicability of the task to bilingual children with diverse L1-backgrounds. It should be; however, noted that most of the studies on LITMUS-SRT report lower-cut-off scores separating TD from SLI in the bilingual groups, and that the task can only be used to assess bilinguals who had at least 12 months of exposure to the L2 (see Tuller et al., 2018).

Non-word repetition belongs to the core assessment measures used for diagnosing LI and has been identified as a reliable clinical marker of SLI in monolingual children (Conti-Ramsden et al., 2001; Gathercole, 2006). An advantage of NWR over other language measures is that it is less affected by prior knowledge of vocabulary and morphosyntax (Thordardottir and Brandeker, 2013; Chiat, 2015) and counts as a relatively culturally fair measure, which could be used for the assessment of children with diverse linguistic and socio-economic backgrounds (Engel et al., 2008; Chiat and Polišenská, 2016). As such, NWR tasks offer promising tools for the identification of SLI especially in bilingual children with limited exposure to the L2.

Measured by increasing numbers of syllables, NWR has traditionally been used to assess phonological working memory (Archibald and Gathercole, 2007; Coady and Evans, 2008). However, the ability to repeat non-words does not only rely on phonological working memory but also requires phonological skills like speech perception, phonological encoding, storage and retrieval of phonological representations, phonological assembly and articulation, which also relate to the capacity of learning new words (Gathercole, 2006). Each of these skills can be deficient in language-impaired children (Coady and Evans, 2008; Marshall, 2014). Recent studies have shown that children with SLI are not only sensitive to the amount of phonological material, i.e., number of syllables in the non-words, but also to phonological complexity such as the presence of consonant clusters, which comprise a particular source of difficulty for children with (phonological) SLI in many languages (Barlow, 2001; Gallon et al., 2007; Marshall and van der Lely, 2009; Ferré et al., 2012; Tamburelli and Jones, 2013; Leonhard, 2014).

Designing an NWRT that identifies LI in bilingual children without disadvantaging those with less experience with the L2 is not straightforward. Despite being less reliant on LS knowledge, there is substantial evidence that performance on NWR (both within and across languages) is affected by the characteristics of the non-words such as word-likeness, length, complexity, prosodic structure, phonotactic probability, and neighborhood density. For instance, children are found to perform significantly better on non-words that are more wordlike, carry LS stress patterns, contain LS-morphemes or have higher phonotactic probability (Jones et al., 2010; Messer et al., 2010; Leclercq et al., 2013; for an overview see Chiat, 2015). These findings imply that “experience and knowledge of lexical phonology contribute to NWR” (Chiat and Polišenská, 2016), which, depending on the nature of the non-words, is generally shown to relate to vocabulary size in monolingual (Gathercole, 2006) and bilingual children (e.g., Engel de Abreu et al., 2013). Departing from that, different LITMUS-NWRTs manipulating factors shown to influence performance on NWRTs such as length, prosody and/or syllable complexity were constructed within the COST IS0804 framework for NWR (see Chiat, 2015 for details).

Similar to the LITMUS *Crosslinguistic* (Quasi-Universal) NWR test (CL-NWRT, Chiat, 2015), the German LITMUS-NWRT (Grimm et al., 2014) was constructed parallel to the French LITMUS-NWRT (dos Santos and Ferré, 2018) within the COST Action IS0804 framework for NWR tests. Unlike the CL-NWRT, e.g., the Dutch Quasi-Universal NWRT (Boerma et al., 2015; Boerma and Blom, 2017), which primarily tests phonological short-term memory and comprises phonologically simple non-words compatible with the phonological properties of any language, the German LITMUS-NWRT was devised to tap more directly into phonological abilities by focusing on phonological complexity. The latter was found to be a promising marker for assessing phonological impairment (Marshall et al., 2002; Ferré et al., 2012; for German, see Ott et al., 2006). LITMUS-NWRTs of this type systematically vary segmental (articulatory difficulty), syllabic (presence or absence of clusters) and sequential complexity (types of consonant and syllable sequences) combining them into non-words of increasing phonological complexity. At the same time, LS phonological properties are controlled as far as possible to avoid penalizing bilingual children. In order to limit effects of lexical knowledge, the non-words were constructed to be maximally distinct from real words in the target language (German) and were created using elementary blocks (segments and syllables) that are cross-linguistically well-attested (Maddieson et al., 2011). In line with the COST Action IS0804 framework (Chiat, 2015), the latter blocks were combined and manipulated in two sets, a set of phonologically complex items with phonological properties common in most of the world’s languages (the quasi language-independent part, LI_part), and an additional set of items containing the same building blocks of the LI_part in addition to the extrametrical /s/ as a complexity variable specific to German and some other languages (the language dependent^3^ part, LD_part). The maximum non-word length is limited to three syllables in both parts in order to minimize working memory load, which could undermine the effect of phonological complexity. Various studies reported negative effects of language specific properties of the NWRTs on performance of bilingual children resulting in insufficient diagnostic accuracy, e.g., Kohnert et al. (2006), Windsor et al. (2010), Boerma et al. (2015), and Armon-Lotem and Meir (2016). However, since the construction of the LD_Part in the German LITMUS-NWRT varies considerably from other LS NWRTs (see section “The German LITMUS Non-word Repetition Task”), bilingual children are not expected to be disadvantaged by the LD_part of this particular task. Although they might encounter more difficulties with the LD items, both monolingual and bilingual children with SLI are anticipated to disproportionately struggle with the structurally more complex LD items since both SLI groups are assumed to have similar underlying deficits (Paradis et al., 2011a,b). Indeed, studies by Ferré et al. (2015), dos Santos and Ferré (2018), Grimm and Hübner (in press), as well as Abed Ibrahim and Hamann (2017) have pointed to the fact that the structurally more complex LD_part of the NWRT did not disadvantage the BiTD children, who performed on par with their monolingual peers. On the contrary, compared to the LI part, the gap between SLI and TD was larger for the LD_part leading to better diagnostic accuracy in both monolingual and bilingual populations. These results corroborate that phonological complexity is vulnerable to phonological deficits not only in monolingual but also in bilingual children.

Several recent studies (e.g., Armon-Lotem and Meir, 2016; Meir et al., 2016; Meir and Armon-Lotem, 2017; Boerma and Blom, 2017; Tuller et al., 2018; Chilla et al., in press) investigated the diagnostic potential and impact of different variables related to bilingualism on the performance in LITMUS-SRTs and NWRTs. Here, we report on three studies of direct relevance to the present research that were conducted within the joint German-French project (BiLaD) using similar methodology with bilingual groups (Arabic/Portuguese/Turkish as L1) in Germany and France, who vary in their sociolinguistic settings. De Almeida et al. (2017) investigated the diagnostic accuracy of French LITMUS-SRT and NWRT and examined whether factors of L2 language use and exposure had an influence on the bilingual children’s performance. Although both tasks significantly discriminated between SLI and TD in both monolingual and bilingual children, reduced specificity of SRT was observed for children not dominant in French. Significant correlations were found between SRT-performance and language use and dominance in the BiTD but not in the BiSLI group suggesting that dominance might be responsible for the variation observed in the BiTD group. To avoid cases of overdiagnosis and enhance diagnostic accuracy, the authors recommend combining SRT with NWRT, which did not correlate with any of the L2-exposure variables.

Tuller et al. (2018) report on direct comparisons of German and French LITMUS-NWRTs and SRTs. Their results showed good to excellent diagnostic accuracy in monolinguals, whereas the diagnostic accuracy for bilinguals was fair to good, i.e., the tasks generally distinguished bilingual children likely to be language-impaired from those likely to be typically developing. The authors further explored whether performance on the two tasks was mainly ascribed to developmental risk factors for SLI or to factors related to bilingualism. Results show that a sizable proportion of the variance in the performance of the bilinguals (BiSLI and BiTD collapsed together) in the German and French LITMUS-SRTs and NWRTs was explained by risk factors of SLI as measured by the index of Positive_Early_Development (see section “The LITMUS-Questionnaire for Parents of Bilingual Children” for details). Exposure and use variables such as current L2-richness accounted for additional 4% of the variance in the French-SRT and 11% of the variance in the German SRT. For the German NWRT, early L2-exposure weighed negatively to account for a further 7% of the variance. Since current L2-richness and early exposure to L2 both contribute to establishing language dominance based on the PaBiQ (see section “The LITMUS-Questionnaire for Parents of Bilingual Children”), this raises the question of whether language dominance has a negative impact on the diagnostic accuracy of the LITMUS-tools, especially on the LITMUS-SRT.

This question was further pursued in Hamann and Abed Ibrahim (2017), who used k-means cluster analysis to group bilingual children based on their performance scores on German LITMUS-SRT and NWRT as language impaired or not without access to their clinical group membership based on standardized assessment. In order to measure diagnostic accuracy, the children’s k-means cluster membership based on SRT and NWRT scores was compared to the likelihood of a child to have SLI or TD based on standardized assessment in each of the child’s languages (see section “Participants” for details). Whereas the sensitivity rates for both SRT (scored by identical repetition, SRT_Id) and NWRT were excellent, the specificity rates were only suggestive, as several bilinguals were assigned to the clinical cluster based on their global NWRT and SRT_Id scores. In line with previous studies on German LITMUS-SRT, this study showed that using the rating measure “target structure” (SRT_Tar), which focuses on the mastery of the constructions targeted by the task, resulted in better specificity and better overall diagnostic accuracy than SRT_Id in the bilingual groups. The individual scores of the children likely to be BiTD were plotted against language dominance for each of the tasks. While NWRT appeared to be rather unaffected by language dominance; 25% of the L1-dominant children performed below cut-off even on SRT_Tar. Finally, the study showed that a combination of SRT and NWRT helps to avoid cases of over-identification.

Given that assessment of bilingual children is usually exclusively carried out in the societal language, the finding that dominance appears to influence the SRT performance of BiTD children, especially those dominant in their L1, raises concerns whether this task is suited for the identification of SLI in L1-dominant children when administered in their weaker language German. However, the three studies above have their limitations: in all of them, diagnostic accuracy of the tools was measured against established clinical status based on standardized evaluation in the L1 and L2, which does not take into account cases of selective impairment or problems with L1 standardized tests in heritage contexts. This, in turn, might be responsible for the reduced accuracy rates (see de Almeida et al., 2017 and Hamann and Abed Ibrahim, 2017 for a discussion). Hamann and Abed Ibrahim (2017) showed that using an alternative procedure that takes into account selective impairments and problems with L1-assessment in minority contexts minimized the slight overlap between BiTD and BiSLI and enhanced diagnostic accuracy. A further limitation is that in both of de Almeida et al. (2017) and Hamann and Abed Ibrahim (2017), dominance was not factored in as a variable into a regression analysis model and might have been confounded by other variables. Hence, the assumed influence of dominance remains a conjuncture that needs to be statistically validated.

### The Present Study

In line with much recent research and building upon our own research, this study investigates the identification of LI in bilingual populations using sentence and nonword repetition tasks. Since both LITMUS-SRT and NWRT were designed to minimize bias against bilingual populations while being indicative of the presence or absence of LI, the following research questions emerge in the light of previous findings:

i. Upon sufficient exposure to the L2, how robust are German LITMUS-SRT and NWRT against language dominance? Are they only sensitive to risk factors for SLI or could background variables related to bilingualism, in particular the degree of language dominance (estimated by relative amount of use of and exposure to L1/L2), compromise their diagnostic accuracy?
ii. Since a combination of tools evaluating different aspects of language ability such as morphosyntax and phonology is recommended to acknowledge the heterogeneity within the SLI population and avoid cases of over- and underdiagnosis, does a combination of LITMUS-SRT (especially when scored by correct target structure) and NWRT yield higher accuracy rates that those estimated for each of the tasks in isolation?
iii. Does a combination of SRT_Tar, which evaluates the mastery of complex constructions and the phonologically more complex LD part of the NWRT provide better diagnostic accuracy for identifying SLI in (monolingual) and bilingual children than other combinations of measures?

To address these questions, we will use an unsupervised machine learning algorithm, the Partitioning Around Medoids (PAM, Kaufman and Rousseeuw, 2009) for deriving a clinical category (clustering) for the children as ± language-impaired based on their performance scores on SRT and NWRT (in isolation and combined) while withholding information about their clinical status based on standardized assessment in L1 and L2. Subsequently, we will calculate diagnostic accuracy of the tasks (separately and combined) by verifying the goodness of the fit against the clinical groups we can establish for bilinguals by their scores in norm-referenced L1 and L2 tests (see section “Participants”), and use regression analysis to investigate which background variables (age, AoO, LoE, degree of language dominance, SES, and risk factors for SLI) best explained clinical-group-membership based on the children’s NWRT and SRT performance scores. Our premise is that if the PAM-cluster membership can be predicted by the presence of risk factors for SLI but not by any of the other background variables known to influence performance of bilingual children on language tests, particularly the degree of language dominance, then clustering of cases cuts across the SLI/TD dimension confirming that the LITMUS-SRT and NWRT are sensitive to LI and are not biased against bilingual children regardless of their language dominance.

## Materials and Methods

### Establishing Language Dominance in Child Bilinguals

A number of methods have been put forward for measuring and operationalizing language dominance in bilingual children. These measures fall into two categories: performance-based measures and experiential-based measures (Unsworth, 2016; Unsworth et al., 2018). Estimates of language dominance obtained by performance-based measures are based on quantitative differences in proficiency measurements between the two languages of a bilingual. These measures are usually extracted from (a) spontaneous speech data, such as mean length of utterance (MLU), upper bound (UB, length of the longest utterance in a speech sample), multi-morphemic utterances (MMU), lexical diversity measures (number of different word types, verbs, and nouns) and directionality of code-mixing (see Cantone et al., 2008; Kupisch, 2008; Bedore et al., 2012 for an overview), and (b) proficiency measures based on standardized tests for vocabulary and grammar. Experiential measures, on the other hand, rely on biographical information and estimates of language use and exposure to predict dominance in bilingual children. The rationale behind the latter approach is that the (relative) proficiency of bilingual children in each of their languages is “in some sense a function of the amount of language to which they are exposed in these two languages” (Unsworth, 2016, p. 156). Accordingly, experiential variables like the relative amount of language use and exposure can be used as a predictor for the degree of bilingual language dominance.

Bedore et al. (2012), Unsworth (2016) as well as Unsworth et al. (2018) found that relative amount of exposure and use reliably predicted dominance group membership as determined by proficiency measures, confirming that relative amount of use and exposure can be used as a proxy for language dominance in bilingual children. For the purposes of the present study and building upon the findings of Bedore et al. (2012), Unsworth (2016), and Unsworth et al. (2018), we use experiential-based measures to establish language dominance for our participants and calculate this based on the information obtained by the PaBiQ as outlined in “The LITMUS-Questionnaire for Parents of Bilingual Children”.

### The LITMUS-Questionnaire for Parents of Bilingual Children

Bilingual children vary considerably in properties of their language exposure and use, which in turn influence the rate and outcome of their language development (e.g., Gathercole and Thomas, 2009; Chondrogianni and Marinis, 2011; Paradis, 2011; Hoff et al., 2012). Thus, having a clear idea about the relative amount of exposure and use for each of the bilingual child’s languages should help professionals to interpret language performance in L1 and L2 adequately and determine whether a child’s (poor) language performance is linked to possible risk factors for LI or to factors related to bilingualism such as the timing, quality and quantity of exposure to the L1/L2, and degree of language dominance.

In order to gather relevant background information, the Questionnaire for Parents of Bilingual Children (PaBiQ; Tuller, 2015) developed during COST Action IS0804 on the basis of the Alberta Language and Development Questionnaire (ALDeQ, Paradis et al., 2010) and the Alberta Language Environment Questionnaire (ALEQ, Paradis, 2011) was used to interview the parents/legal guardians of the participating children. The parents of participants in the study were interviewed orally in their language of preference by trained native bilingual interviewers familiar with the respective culture.

The PaBiQ incorporates questions about developmental risk factors for SLI, which are synthesized into a global No Risk Index, for which a maximum of 23 points can be attained. This index is arrived at by collapsing the scores of the Positive Early Development index, which is associated with the timing of early language developmental milestones, and the Family History index, which is associated with the presence of oral/written language disabilities in the family. The Positive Early Development index (/14 pts) is calculated by adding up the sub-scores for age of first word (≤15 mo = 6 pts; 16–24 mo = 4 pts; >25 mo = 0 pts), age of first multiword utterances (≤24 mo = 6 pts; 25–30 mo = 4 pts; >31 mo = 0 pts) and early parental concerns (yes = 0 points; no = 2 points). The familiar risk for SLI (/9 pts) is indexed by the existence of first-degree relatives (mother, father, siblings) with reading/writing problems, difficulties understanding others when they speak or difficulties expressing themselves orally. Children with a negative family history of language problems are awarded a maximum of 9 points (3 × 3: 1 point per family member per type of language difficulty). Boerma and Blom (2017) investigated the influence of LI and bilingualism on the latter two indices and looked into their diagnostic accuracy. In line with Paradis et al. (2010), they reported strong negative effects of LI on Early Language Development and showed that it was a strong predictor of LI in both monolingual and bilingual children confirming previous findings that a late onset of first words and sentences in at least one language is a risk factor for SLI (cf. de Houwer, 2009; Reilly et al., 2010). With regard to the Family History index, Boerma and Blom (2017) observed a negative effect for LI in the monolingual group but not in the bilingual one and concluded that, due to cultural factors, “Family History as reported by parents may […] be less reliable as an index of LI in bilingual children than in monolingual children” (p. 73). The Positive Early Development Index also yielded promising diagnostic results in the study by Tuller et al. (2018), who found it be the leading factor explaining performance differences between BiSLI and BiTD in both of the German and French LITMUS non-word and sentence repetition tasks.

**FIGURE 1:**
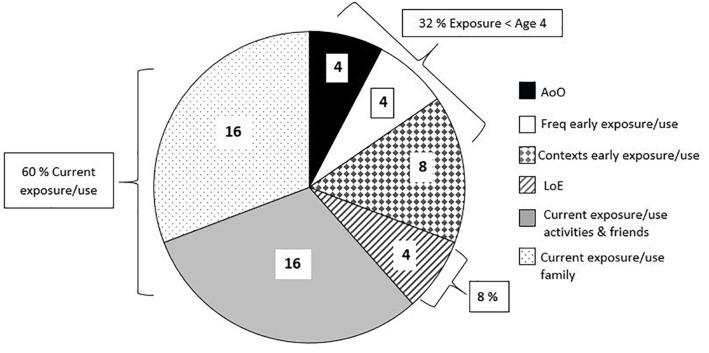
PaBiQ: Calculation of language exposure index.

The PaBiQ further allows the calculation of a Language Dominance Index (LDI) as a differential between the L1 Exposure Index (relative amount of exposure to the L1) and the L2 Exposure Index (relative amount of exposure to the L2, i.e., German). For each of the child’s languages a total of 50 exposure/use^4^ points could be attained using the German PaBiQ^5^. The Exposure Index is calculated for each of the child’s languages separately based on AoO, LoE^6^, frequency of early language use and exposure^7^, i.e., before the age of four, language richness before the age of four as measured by diversity of language exchange contexts, current language exposure/use within the family, current language use/exposure during different activities within an average week and in exchanges with playmates and family friends. The latter composite score also counts as an estimate of current language richness. An Exposure Index (/50 points) for L1 and L2 emerges by adding up the aforementioned sub-scores. A visual representation of the relative contribution of each of the sub-scores toward establishing the Exposure Index is given in Figure 1. As can be seen in Figure 1, current language use/exposure contributes the lion share (60%) to the calculation of the Exposure Index and consequently the LDI. This converges with the findings of Bedore et al. (2012) in their large-scale study, in which estimates of current language use (a composite score based on children’s amount of exposure and language output) accounted for 60% of the variance in language dominance patterns of bilingual children.

The language dominance index is then obtained by subtracting the L1 Exposure Index from that of the L2 yielding an estimate of the child’s degree of L2-dominance on a scale from -50 (extremely dominant in the L1) to +50 (extremely dominant in the L2). De Almeida et al. (2017, p. 5) compared multiple LDI cut-offs around LDI = 0 (optimal balanced bilingual) against impressions of bilingual investigators of the individual children after interacting with them and their families in both of their languages, and defined cut-off points for language dominance in attempt to explore the use of this variable. An LDI between -5 and +5 was set as a cut-off separating dominant from balanced bilinguals. Children with LDIs ranging from -5 to +5 are classified as “balanced,” children whose LDI is below -5 are considered to be dominant in the home language, while children with an LDI above +5 are classified as dominant in the societal language German.

The questionnaire further allows determining the family’s socio-economic status (SES) based on the mother’s and the father’s educational levels. For the purposes of the current paper, maternal rather than paternal educational level (as measured by years of education of the mother) is used as a metric for SES, since the former is reported to be a strong predictor of language development, especially for expressive vocabulary levels, in both monolinguals (Hoff, 2003, 2006) as well as child bilinguals (Paradis, 2009; Calvo and Bialystok, 2014; Paradis and Jia, 2016; Meir and Armon-Lotem, 2017). SES-related language deficits^8^ are reported to have a negative effect on performance in tasks with rich linguistic load, e.g., SRTs and NWRTs with word-like items (Roy et al., 2014; Chiat and Polišenská, 2016).

### The German LITMUS Sentence Repetition and Non-word Repetition Tasks

#### The German LITMUS Sentence Repetition Task

The German LITMUS-SRT (Hamann et al., 2013) used in this study was constructed in close parallel to the French LITMUS-SRT (de Almeida et al., 2017; Fleckstein et al., 2018). It consists of 45 sentences divided in three levels of syntactic complexity (five conditions per level controlled for syllable number, three test items per condition). The degree of an item’s structural complexity relies on the presence of syntactic operations such as Wh-movement, clausal embedding, intervention^9^ – where the latter may add difficulty to the presence of two propositions. Accordingly, level 1 consists of simple declaratives (7–9 syllables) and focuses on Subject-Verb-Agreement (SVA), tense and the sentence bracket[see (1)]. Level 2 (9–13 syllables) includes two types of object questions: bare Wh-questions with the non-D-linked *wh*-operator (Wen “who-*masc.-acc.*”), and *Which* NP-questions with the discourse-linked *wh-*operator (*Welchen* “which-*masc.-acc.*”) followed by an intervening lexical noun phrase [see (2a) & (2b)]. Bare Wh-questions are considered to be structurally less complex since they do not involve intervention. Level 2 further contains non-finite and finite [see (3)] complement clauses. The latter are contrasted with coordinate structures, which serve as control items (two propositions but no embedding). Level 3 (11–12 syllables) comprises the most complex constructions and tests long passives, topicalizations [see (4)] as manifestations of the V2-property^10^ of German, subject relative clauses as well as object relative clauses with [see (5)] and without intervening lexical determiner phrases.

Note that German has morphological case marking on accusative masculine singular pronouns, such as the interrogative and relative pronouns in examples 2a, 2b, and 5. Table 1 gives an overview of test conditions. For more details on German LITMUS-SRT, we refer to Hamann et al. (2017) and Hamann and Abed Ibrahim (2017).

**Table 1:** German LITMUS-SRT: Overview of test conditions.

Level 1	Level 2	Level 3
SVO-Present	Object who-question (bareWH)	Passive
SVO-Simple past	Object which-question (Wh-NP)	Topicalization
Sentence bracket (Aux)	Coordination (Coord)	Subject relatives (SR)
Sentence bracket (Particle)	Non-finite complement Cl	Object relatives without intervener (OR –intv.)
“Werden” control	Finite complement Cl (CompFin)	Object relatives with intervener (OR+intv.)

(1) *Sentence bracket*:
  Der Prinz **hat** die Prinzessin **umarmt**
  The/*nom.* prince has the/*acc.* princess hugged
  “The prince hugged the princess”
(2a) Bare WH
  **Wen** beißt der große Löwe immer?
  Who/*acc.* bites the/*nom.* big lion always?
  “Who(m) does the big lion always bite?”
(2b) Which-NP
  **Welchen Bauern** ärgert der Affe?
  Which/*acc.* peasant annoys the/*nom.* monkey?
  “Which peasant does the monkey annoy?”
(3) Finite complement clause:
  Der Wikinger glaubt, **dass** die hexe ihn **mag**.
  The/*nom.* viking believes, that the/*nom.* witch him likes
  “The viking believes that the witch likes him”
(4) Topicalization
  **Den Arzt** fotografiert der Bauer gerne
  The*/acc*. doctor photographs the/*nom.* peasant gladly
  “The doctor, the peasant photographs gladly”
(5) Object relative with intervention:
  Ich sehe den Vogel, **den der Pinguin** weckt.
  I see the/*acc*. bird who/*acc.* the/*nom.* penguin wakes up
  “I see the bird who(m) the penguin wakes up”

The test stimuli are pre-recorded, pseudo-randomized and integrated into a child friendly PowerPoint Presentation. The administration of the task takes about 10 minutes. The task is scored both by identical repetition of test items (SRT_Id), i.e., whole item accuracy, where only phonological errors are disregarded, and by correct target structure (SRT_Tar), which measures whether a particular structure has been mastered or not (see Marinis and Armon-Lotem, 2015 for scoring measures). Although scoring by SRT_Id is faster and easier, L2-errors not affecting the realization of the targeted structure such as lexical substitutions, omissions and systematic recurrent case^11^ as well as gender errors could surface using this scoring method and penalize bilingual children. Comparison of these scoring methods has indeed shown that SRT_Tar leads to higher diagnostic accuracy of the test for German (see Hamann and Abed Ibrahim, 2017 for particulars).

#### The German LITMUS Non-word Repetition Task

The German LITMUS-NWRT (Grimm et al., 2014) employed in this study is composed of two parts: a structurally less complex (quasi-) language independent part (NWRT_LI) and a language dependent part (NWRT_LD) incorporating more complex structural aspects. In both parts the item length ranges from one to three syllables with constant word-initial stress. The 30 items of the LI part were constructed using phonemes and phonotactic constraints attested in the vast majority of the world’s languages (Maddieson et al., 2011), i.e., phonemes that are “compatible with cross-linguistically diverse constraints on lexical phonology” (Chiat, 2015, p. 138). Unlike the non-words of the Quasi-Universal-NWRT discussed in Chiat and Polišenská (2016), the non-words of the German LITMUS-NWRT are shorter and are not only composed of simple CV sequences, but also include syllables with initial consonant clusters “#CCV” or closed syllables of the type “CVC#,” which are typologically well-attested albeit their relative complexity (Maddieson, 2006). Throughout the task, phonological complexity is systematically varied at the segmental (consonantal), syllabic (presence of branching onsets or coda) or sequential (position of cluster within the non-word) levels (see dos Santos and Ferré, 2018; Grimm and Hübner, in press for details). The LD part contains 36 items adhering to the same construction principles of the LI part in addition to the extrametrical /s, ʃ/ in word initial and final positions as a complexity feature specific to German (and some other languages, e.g., English and Russian). Such sC sequences violate the Sonority Sequencing Principle and are considered phonologically more complex than other types of onset clusters. Constructed as such, the LD_part is considered to be structurally more complex compared to the LI_part, yet less dependent on LS knowledge than the more traditional Language-Specific NWRTs, e.g., Rispens and Baker (2012), which draw on the full phoneme inventory (consonants and vowels) and include many more properties specific to the target language (Chiat, 2015; Chiat and Polišenská, 2016).

Although structures with higher phonological complexity are generally more error-prone in TD children, they are “disproportionately difficult” for children with SLI (Chiat, 2015, p. 137), who struggle with phonological complexity (Archibald and Gathercole, 2006; Jones et al., 2010; dos Santos and Ferré, 2018). Thus, a greater performance gap between TD and SLI is expected for both monolingual and bilingual children on NWRT_LD, which contains trilateral sCC onset clusters, where /s/ and /ʃ/ represent an appendix to the prosodic word. The latter has been shown to be deficient in phonologically impaired monolingual German children (Ott et al., 2006). An overview of segments and syllable types is given in Table 2.

**Table 2:** Overview of segments and syllable types in German LITMUS-NWRT.

	Vowels	Consonants	Syllable types	Examples
Language-	/a, i, u/	/p, k, f, l/	CV	kapi
Independent part			CCV	plaklu
(LI)			CVC#	pukif
30 items				
23 test items				
7 controls (e.g., faku, paf)				
Language-Dependent part (LD)	/a, i, u/	/p, k, f, l/	same syllable types plus	
36 items		plus	#sCV	sfikupla
32 test items		/s/	#sCCV	sklipafu
4 controls (e.g., kiʃ, sapi)		/ʃ/	Cs# internal /s/	kapifaps fikuspa


Task administration takes about 5 min and the non-words are presented to the child in a pseudo-randomized order via an animated PowerPoint Presentation. At the beginning of the task, children are provided with noise-canceling headphones and are told that an alien from another planet would appear on the screen and try to teach them his language (format adapted from Engel de Abreu et al., 2013). The test is scored by whole item accuracy (percentage of items correct), since this scoring method is better suited for clinical purposes and has been shown to be informative (Roy and Chiat, 2004; Boerma et al., 2015). A response is rated as correct if all consonants and vowels in addition to their sequencing correspond to the target form. Phoneme omissions, substitutions or additions are regarded as incorrect. Systematic phoneme replacements reflecting articulatory difficulties, e.g., /t/ for /k/ (/kafip/→/tafip/) are not counted as errors. Since the task mainly targets bilingual children, L2-errors such as voicing of consonants (/pilu/→/bilu/) or vowel alternations (/faku/→/fako/) are disregarded. Furthermore, substitution of extrametrical /ʃ/ through [s] or an interdental pronunciation of extrametrical /s/ are not counted as errors since this does not result in a phonemic contrast in extrametrical positions in German (Grimm and Hübner, in press).

### Participants

The present study was conducted in line with the compliance form, transaction number 20120416505890730506, of the German Science Foundation and the recommendation of the “Kommission für Forschungsfolgenabschätzung und Ethik” (commission for the evaluation of research consequences and ethics) of the Carl-von-Ossietzky University of Oldenburg (rf. Drs. 21/16/2013). Parents or legal guardians of all participating minors provided written informed consent for both data collection and analysis. The research protocol was approved by the “Kommission für Forschungsfolgenabschätzung und Ethik” of the Carl-von-Ossietzky University of Oldenburg.

Except for 3 children, the current study used the same participant sample as Hamann and Abed Ibrahim (2017), including 77 children, 21 German monolinguals and 56 L2-German bilinguals with Arabic, European Portuguese or Turkish as L1. The latter L1s were chosen because a sizable proportion of immigrants residing in Germany are of Arab, Portuguese and Turkish origin. Furthermore, the typological differences between them and the children’s L2 (German) enable cross-group comparisons, e.g., Abed Ibrahim et al. (2018) and Chilla et al. (in press). The age range of the participants was 5;6–9;0 years covering the last year of kindergarten and the crucial first 2–3 years of primary school. As inclusion criteria for bilingual children, children had to have a minimum L2 exposure of 18 months and be at least functionally bilingual. Thus, children who failed to complete even receptive subtests in the L1 were excluded from the study. 49/56 children were simultaneous bilinguals, while 7 were sequential bilinguals, whose systematic exposure to L2 mainly started upon kindergarten entry at approximately age three. Almost all of the bilingual participants had a LoE to German of more than 24 months at the time of testing with a mean LoE of 5;1 years (*SD* = 1;10). Children likely to have SLI, i.e., with a clinical diagnosis of SLI, were recruited from specialized speech-language pathology centers and kindergartens with special inclusion programs from different parts of Germany. Given the high rates of over- and under-referral of bilingual children to speech language therapy (Grimm and Schulz, 2014), an extensive procedure based on standardized evaluation in each of the child’s languages was applied in order to verify the clinical status of all recruited bilingual children as ± language-impaired. The verification of clinical status was done in accordance with the recommendations of the COST Action IS0804 assessment committee as outlined in Thordardottir (2015, p. 343) and began with a control for non-verbal intelligence using the German version of Raven’s Colored Progressive Matrices (CPM; Bulheller and Häcker, 2002). Only Children who had a non-verbal IQ score ≥ 80 were included in the study. In addition to standardized assessment, narrative samples were collected from each child in both of her languages using the picture materials provided by the LITMUS-Multilingual Assessment Instrument for Narratives (MAIN, Gagarina et al., 2015). The collection of the narrative samples was done in accordance with the MAIN protocol (story telling). However, for the purposes of the current study, the latter samples were not analyzed in terms of narrative macro- and microstructure, but were rather used as spontaneous speech samples. Especially in borderline cases, the latter samples were consulted in order to gain an impression^12^ about the child’s expressive language abilities in both of her languages and look for clinical markers for SLI, e.g., SVA errors, the use of infinitives and verb placement errors in German (Clahsen, 1991; Rice et al., 1997; Hamann et al., 1998; Lindener, 2002).

As to assessment using formal tests, in our previous work, e.g., Hamann and Abed Ibrahim (2017), Tuller et al. (2018), and Chilla et al. (in press), we adapted the criteria outlined in Leonard (2014) to bilinguals using Thordardottir’s (2015) recommendations and assigned a child to the BiSLI group if she scored below dominance-adjusted^13^ norms in two language domains (on norm-referenced tests) in both of her L1 and L2. Five language areas relevant in this context were evaluated in each of the child’s languages (except for Turkish): phonology, morphosyntax comprehension and production as well as receptive and expressive vocabulary (see also Tomblin et al., 1996). Since expressive vocabulary is a notorious locus of difficulty for bilingual children, we counted lexicon as a single domain and considered the child unimpaired in this domain if only receptive vocabulary was above the respective cut-off. For the assessment of L1 and L2, we chose norm-referenced L1 and L2 tests frequently used by speech language pathologists and cover the age range^14^ under investigation (see Table 3 for a detailed overview of standardized assessment tools). For German, we selected the LiSe-DaZ (Schulz and Tracy, 2011), which provides bilingual and monolingual norms, for assessing morphosyntax. The short form of the WWT (Glück, 2011) was used to assess receptive and expressive vocabulary, and the screening version of the PLAKSS-II (Fox-Boyer, 2014) was used to evaluate phonology. We tried to assess the same language domains in Arabic, Portuguese and Turkish. For Arabic, this was possible using the comprehensive test battery ELO-L (Zebib et al., 2017), which offers norms for Lebanese Arabic and was adapted to a number of other varieties of Arabic^15^ by the test authors in collaboration with linguistically trained native speakers of the respective varieties (Algerian, Iraqi, Libyan, Moroccan, Palestinian, Syrian, and Tunisian). We used the PALPA-P test battery (Castro et al., 2007) for Portuguese. One major limitation of the PALPA-P is that it lacks norms for some of the age ranges we are investigating for the lexical domain. As a result, we chose to assess receptive and expressive vocabulary using subtests of the GOL-E (Sua-Kay and Santos, 2014), which covers our entire age range, and used subtests of the PALPA-P to assess phonology and morphosyntax. For Turkish, we chose the TEDIL (Topbaş and Güven, 2013), which measures morphosyntactic comprehension and production as well as lexical semantics. The test; however, does not include a subtest for phonology and does not offer norms for the individual subdomains. Instead, a composite score exists for each of comprehension and production collapsing morphosyntax and lexical semantics together. As the Turkish test merely offers a single production and a single comprehension score, encompassing two domains each, a child was assigned to the BiSLI group if she scored below cut-off in either production or comprehension. For a detailed description of standardized assessment L1-L2-tests and a complete overview of recruitment and classification procedure of bilingual children into TD vs. SLI, we refer to Hamann and Abed Ibrahim (2017).

**Table 3:** Overview of norm-referenced tests employed for standardized language assessment in Arabic, German, European Portuguese, and Turkish.

Language	Test	Language skill evaluated					Method of scoring	Age rage
				
		Phonology	Reception vocabulary	Expression vocabulary	Morphosyntax comprehension	Morphosyntax production		
Arabic	ELO-L^a^	Word repetition	Picture selection	Picture naming	Picture-sentence matching	Sentence completion	Individual subtest scores	3;0–7;11
German	WWT 6–10^b^	–	Picture selection	Picture naming	–	–	Individual subtest scores	5;6–10;11
	LiSe-DaZ^c^	–	–	–	Picture-sentence matching, TVJT	Story, sentence completion, lead-in questions	Individual subtest scores	Monolinguals: 3;0—6;11 Bilingual: 3;0–7;11
	PLAKSS-II^d^	Picture naming	–	–	–	–	Individual subtest scores	2;6–7;11
European Port.	PALA-P^e^	Non-word repetition	Picture selection	Picture naming	Picture selection	Sentence repetition	Individual subtest scores	5;0–9;11 (with missing norms for some age ranges for all tasks)
	GOL-E^f^	–	Word definition	Antonyms naming	–	Complex S from two simple S‘s	Individual subtest scores and global score	5;07–10;00
Turkish	TEDIL^g^	–	Picture selection	Picture naming	Picture Selection	Sentence completion/ constrcution	2 composite scores, 1 production and 1 comprehension	2;0–7;11


*^*a*^Zebib et al. (2017); ^*b*^Glück (2011); ^*c*^Schulz and Tracy (2011); ^*d*^Fox-Boyer (2014); ^*e*^Castro et al. (2007); ^*f*^Sua-Kay and Santos (2014); and ^*g*^Topbaş and Güven (2013); TVJT, truth value judgment task.*

Following the argumentation in Hamann and Abed Ibrahim (2017, p. 16) about problems encountered with standardized L1 tests in heritage contexts, and since our previous classification procedure did not isolate subgroups of SLI and might have missed cases of selective impairment such as grammatical/syntactic, phonological or lexical SLI (cf. Friedmann and Novogrodsky, 2008), we adopted Hamann and Abed Ibrahim’s (2017) modified “criteria for the identification of the bilingual clinical group” in this paper. Accordingly, we assigned a child to the BiSLI group if she had a selective impairment in the L2, i.e., if she performed below the dominance-adjusted cut-off in either morphosyntax or receptive vocabulary or phonology (not necessarily two domains in combination), and scored below norms in two domains in her L1 (one domain for Turkish) or showed poor performance of spontaneous production in both of her L1 and L2. Table 4 gives a participant overview based on clinical status as verified by the modified procedure described above and also includes the two monolingual control groups MoSLI and monolingual typically developing children (MoTD). By applying the modified classification criteria, the clinical status of 4 children who were initially classified as BiTD in Hamann and Abed Ibrahim (2017) changed to BiSLI^16^. In Table 4, the BiTD children are divided into subgroups based on their L1: Arabic = BiTD-A, Portuguese = BiTD-P and Turkish = BiTD-T. The BiSLI group is composed of 12 children (4 with L1 Arabic, 3 with L1 Portuguese and 5 with L1 Turkish). Due to the relatively small sample size, the BiSLI children are grouped together regardless of their home language. The bilingual children are further classified according to language dominance as measured by the PaBiQ (see section “The LITMUS-Questionnaire for Parents of Bilingual Children”). As can be seen in Table 4, almost half of the children in the BiTD group (21/44) are dominant in their L1, whereas the majority of the BiSLI children (9/12) are either balanced or L2-dominant^17^.

**Table 4:** Participants including monolingual controls and bilinguals after verification of clinical status (Mean, *SD* and range^16^).

Simult./total	MoTD (*n* = 10)	MoSLI (*n* = 11)	BiTD (*n* = 44)				BiSLI (*n* = 12)
				
			BiTD-A (*n* = 10)	BiTD-P (*n* = 18)	BiTD-T (*n* = 16)	Total (*n* = 44) 38/44	11/12
Age at testing	75.90	79.00	88.60	83.44	86.68	85.6	80.66
(in months)	(8.99)	(9.89)	(13.5)	(14.72)	(13.01)	(13.60)	(15.05)
	66–92	68–98	70–108	66–108	70–104	66–108	64–108
Age of onset			39	17.61	25.31	25.27	16.00
(in months)	0	0	(13.61)	(24.39)	(15.52)	(20.65)	(17.03)
			24–75	0–90	0–48	0–90	0–36
Length of exposure	75.90	79.00	53.2	67.97	61.37	61.73	62.00
(Gr.) (in months)	(8.99)	(9.89)	(19.68)	(24.59)	(19.01)	(21.87)	(22.77)
	66–92	68–98	32–97	18–101	34–96	18–101	30–88
CPM (PR)	81.20	53.72	44	73.77	71.18	66.06	55.08
	(13.98)	(24.50)	(29.13)	(17.69)	(22.23)	(25.01)	(26.69)
	56–100	25–99	9–93	42–94	38–100	9–100	27–98
LDI (/50)			-7	1.44	-5.75	-3.09	0.58
	N/A	N/A	(9.34)	(13.76)	(12.57)	(12.77)	(12.37)
			-25–11	-25–23	-27–18	-27–23	-21–24
L1-dominant (no./total)	N/A	N/A	(5/10)	(8/18)	(8/16)	(21/44)	(3/12)
Balanced (no./total)	N/A	N/A	(1/10)	(3/18)	(4/16)	(8/44)	(5/12)
L2-dominant (no./total)	N/A	N/A	(4/10)	(7/18)	(4/16)	(15/44)	(4/12)
Yrs. educ. mother			14.2	13	13.68	13.52	11.83
	N/A	N/A	(1.68)	(3.91)	(5.23)	(4.05)	(2.75)
			12–18	4–18	5–20	4–20	8–16


*^*16*^When applicable.*

The four groups (MoTD, MoSLI, BiTD, and BiSLI) were comparable in terms of non-language variables such as chronological age and non-verbal intelligence. Concerning age, the overall effect of Group was not significant, as revealed by Kruskal–Wallis test [*χ^2^*(3, *N* = 77) = 5.505, *p* = 0.138, η*^2^* = 0.034]. This also holds when the BiTD group is split into three subgroups by L1 [*χ^2^*(5, *N* = 77) = 6.758, *p* = 0.239, η*^2^* = 0.051]. In terms on non-verbal intelligence, the overall effect of Group was significant [*χ^2^*(3, *N* = 77) = 8.448, *p* = 0.038, η*^2^* = 0.075]. However, subsequent pairwise comparisons using Mann–Whitney U tests controlling for false positives, that is Type I error, revealed only one marginally significant comparison, namely MoSLI vs. MoTD (*U* = 19.00, *p* = 0.06, *r* = 0.553, Bonferroni-corrected). Yet, all of the children belonging to the MoSLI group have normal non-verbal intelligence. We further checked whether the bilingual groups were comparable concerning SES, AoO, LoE, and degree of L2-dominance (LDI). No significant differences emerged between BiTD and BiSLI concerning SES [*χ^2^*(1, *N* = 56) = 2.228, *p* = 0.135, η*^2^* = 0.041], AoO [χ^2^(1, *N* = 56) = 3.261, *p* = 0.071, η*^2^* = 0.059], LoE [*χ^2^*(1, *N* = 56) = 0.615, *p* = 0.433, η*^2^* = 0.011], and LDI [*χ^2^*(1, *N* = 56) = 1.912, *p* = 0.167, η*^2^* = 0.035]. This also holds when the BiTDs are split by L1 SES [*χ^2^*(3, *N* = 56) = 3.216, *p* = 0.360, η*^2^* = 0.06], LoE [*χ^2^*(3, *N* = 56) = 3.640, *p* = 0.303, η*^2^* = 0.07] and LDI [*χ^2^*(3, *N* = 56) = 4.457, *p* = 0.216, η*^2^* = 0.08. With respect to AoO, the overall effect of Group was significant when BiTDs were divided by L1 into three subgroups L1 [*χ^2^*(3, *N* = 56) = 11.833, *p* = 0.008, η*^2^* = 0.17]. Mann-Whitney U tests applying Bonferroni-adjustment of *p-*values revealed significant differences in AoO between BiTD-A and BiTD-P (*U* = 33.00, *p <* 0.05, *r* = 0.531) as well as between BiTD-A and BiSLI (*U* = 17.00, *p <* 0.05, *r* = 0.617). Nevertheless, the overall effect of Group was not significant when the BiTD groups were collapsed together [*χ^2^*(1, *N* = 56) = 3.261, *p* = 0.071, η*^2^* = 0.059].

### Data Analysis

The children’s responses on the SRT and NWRT were recorded using special dictaphones. Data transcription, verification and coding for errors were done offline by two independent linguistically trained raters (percentage of agreement was at least 90%). For each repetition measure, the percentage of correct responses was used as basis for data analysis. Null reactions were counted as errors, unless they were due to technical problems or errors by the investigators (missing data, less than 1% of the overall data).

IBM SPSS 24 (2016) and R-Studio (2012) were used to conduct statistical analyses. Non-parametric tests were used for group comparisons due to unequal sample sizes and the violation of the normality assumption, checked by the Shapiro-Wilk test. Since we wanted to investigate whether the LITMUS repetition tools are suitable for assessment of bilingual children in their weaker language, we first checked for group differences between L1-dominant BiTDs and their monolingual, balanced and L2-dominant TD peers, and whether performance of L1-dominant BiTDs overlapped with that of MoSLIs and BiSLIs. Here, we split the BiTDs into three subgroups based on LDI as established in the section “The LITMUS-Questionnaire for Parents of Bilingual Children”.^18^ and ran Kruskal-Wallis tests and Mann-Whitney U tests with Bonferroni-adjustment. Recall that BiSLIs were collapsed into a single group due to the small sample size. Since performance of BiTDs on SRT appeared to be influenced by dominance, we ran partial correlation analysis controlling for age on their SRT_Id and SRT_Tar. In addition to language dominance, we also checked for correlations with AoO, LoE and SES, since they are factors known to influence performance on linguistic tasks. Next, linear regression models for predicting performance of the BiTDs on SRT_Id and SRT_Tar were built using the variables that yielded significant correlations.

Secondly, we applied cluster analysis to the data in order to automatically group the children into ± language-impaired based on their performance scores on the SRT (SRT_Id, SRT_Tar) and NWRT (NWRT_global, NWRT_LI, NWRT_LD), separately and then in combination. A clustering algorithm classifies a dataset into several meaningful homogenous sub-categories - so-called clusters (i.e., TD vs. SLI in this study) - based on the values of their attributes (i.e., linguistic variables in the present study) such that the similarity^19^ among objects within a category is larger than that between categories. We opted for unsupervised learning (cluster analysis) for verifying diagnostic accuracy and establishing cut-off points separating TD from SLI on the tasks, since it does not use predefined clinical status during the statistical analysis, and is thus unbiased by any given classification of participants.

Because children were measured based on performance scores on LITMUS-SRT and NWRT designed to identify SLI without penalizing bilinguals, our premise was that SLI-cases would be similar to each other, and hence group together, while TD-cases would form their own cluster regardless of bilingualism. Different from Hamann and Abed Ibrahim (2017), we chose the PAM (Partitioning Around Medoids) non-hierarchical k-medoid clustering method (Kaufman and Rousseeuw, 1987, 2009) over k-means, because it is a suitable method for small datasets with up to approximately 60 objects, and because it can handle noisy data and outliers (Kaufman and Rousseeuw, 1987, 2009; Kashef and Kamel, 2008; Patel and Singh, 2013; Soni and Patel, 2017). Variables were scaled for normalization purposes in the course of the PAM-analysis. We used the function *pam* of the *cluster* R package (Maechler et al., 2017).

We used *Hopkins statistic* (H) based on the *factoextra* R package (Kassambara and Mundt, 2017) as a measure of cluster tendency to assess clusterability (Hopkins and Skellam, 1954). If the H-value is close to zero, and far below 0.5, then the dataset is clusterable (Kassambara and Mundt, 2017; Krishna et al., 2018). Because H is run on the created random dataset every time, we get fluctuations in the H-values if we run the statistics multiple times. Banerjee and Davé (2004) demonstrate that random data sets, clustered data sets and regularly spaced data sets show H-values of around 0.5, 0.7–0.99 and 0.01–0.3, respectively.

Because the k-medoid algorithm requires that the number of clusters should be pre-defined, we first ran the *Gap Statistic* (Tibshirani et al., 2001) to determine the optimal number of clusters. The *Gap Statistic* compares the change in within-cluster dispersion for each clustering solution (at each number of clusters) to that expected at random distribution. We used the functions *fviz_nbclust* of the *Factoextra* R package (Kassambara and Mundt, 2017) and *NbClust* of the *NbClust* R package (Charrad et al., 2014) to determine the optimal number of clusters.

The k-medoid algorithm selects one of the members of the cluster as the most representative object, named *cluster medoid*, so that each cluster has only one medoid. By choosing an actual case (i.e., an SLI or a TD child) as the cluster medoid, the k-medoid method is less sensitive to outliers, as mentioned before. The optimal cluster is achieved by minimizing the sum of squared Euclidean distances to the medoid in each cluster, also called the error sum of squares (Kaufman and Rousseeuw, 1987). First, in the so-called “Build-step,” the k-medoid algorithm selects k medoids randomly, with k being the optimal number of clusters. Next, a matrix of dissimilarity is calculated from the raw data and the algorithm assigns every object to either of the k clusters based on their distance to the nearest medoid (Patel and Singh, 2013). The sum of absolute error in the clustering procedure is equal to the sum of the distances between data points and their medoids. In the so-called “Swap-step,” each non-medoid object is tested as a potential medoid within each cluster by checking if the sum of within-cluster distances gets smaller if that object is used as the new medoid. If this is the case, then that configuration is used. The algorithm checks at each iteration step, if the solution is better than the previous one. If the medoids do not change, the algorithm terminates (see Patel and Singh, 2013 for details).

Because the medoid of each cluster can be seen as a prototype of that cluster, identifying the medoid can serve as a cue to interpret the cluster. For example, if the medoid of a cluster was originally diagnosed as an SLI-case, then that cluster represents most probably the SLI-cases. We expected the SLI-cluster to contain the majority of the children classified originally as SLI based on standardized assessment, while the majority of TD-cases would reside in the other larger cluster. Our further premise was that the cluster with the lower scores on the linguistic variables would represent the cluster with LI, since language-impaired cases score lower on the linguistic variables.

After clustering the sample, we determined the estimated cut-offs on the linguistic variables (i.e., SRT and NWRT) between the SLI- and TD-clusters based on the clustering result. A cut-off is a value of a variable which can be seen as the best threshold score to separate the cases belonging to the two categories using that variable. If the two categories can be best separated along multiple variables simultaneously, e.g., SRT and NWRT combined, then cases can be predicted (as TD vs. SLI in our study) based on multiple cut-offs on these variables. To this end, we employed conditional inference tree models (Tagliamonte and Baayen, 2012). Conditional inference trees (ctrees) are non-parametric regression models visualized as decision trees. They are suitable for our dataset because of the presence of high-order interactions among the variables and the overall small sample-size compared to the number of predictors (Levshina, 2015). Besides determining the cut-off for the linguistic variables, ctrees can also give information about the hierarchical structure of the relevant predictors of cluster membership, i.e., about variable importance. For instance, if clustering is based on several linguistic variables such as SRT_Id, SRT_Tar and NWRT_global, decision trees can show which one contributed the most toward predicting cluster membership as TD or SLI. The higher the variable in the hierarchy, the more important it is, with the highest-level variable being the most important. If there are multiple variables in the ctree, then a multi-hierarchy predicts the outcome (i.e., cluster membership as TD or SLI). Ctrees were implemented with the *party* R package using the ctree function (see Hothorn et al., 2006 for details).

In order to address research questions (ii) and (iii), we calculated diagnostic accuracy^20^ for the SRT and NWRT measures separately and combined. Sensitivity and specificity levels were estimated by comparing cluster membership of each of the children as TD vs. SLI as assigned by PAM on the basis of LITMUS-SRT and NWRT results to their clinical status (as established by the standardized assessment procedure described in “Participants”). Sensitivity is determined by the proportion of children with LI identified as such by LITMUS SRT and NWRT or subtests thereof (i.e., assigned to the clinical cluster in our case), while specificity is computed based on the proportion of children with typical language development identified as such by our tests, i.e., assigned to the non-clinical cluster (Oetting et al., 2008; Dollaghan and Horner, 2011). In addition, likelihood ratios^21^ (LRs) were calculated based on the obtained sensitivity and specificity levels. An advantage of LRs is that they are less likely to be affected by variations in the properties of the test sample (Dollaghan and Horner, 2011). LR+, positive likelihood ratio [sensitivity/(1-specificity)], indicates how likely it is that a score below a cut-off criterion to be present in language-impaired children, whilst an LR-, negative likelihood ratio ((1-sensitivity/specificity), is indicative of the likelihood of a score above a cut-off criterion to belong to a child without LI.

To answer research question (i), we investigated which of the background information variables provided by the PaBiQ as cogent confounders predicted cluster membership following each clustering procedure based on SRT and NWRT measures or combinations thereof. The hypothesis to be tested was that cluster membership as TD or SLI based on performance scores in LITMUS-NWRT and/or SRT can only be explained by variables concerning risk factors to SLI and not by background information variables related to bilingualism, particularly the degree of language dominance. If this hypothesis is confirmed, then the clustering of the cases cuts across the SLI/TD dimension rather than any of the background information variables unrelated to risk factors for SLI validating that the diagnostic accuracy of the tasks is not compromised by language dominance. To that end, we ran Firth’s Bias-Reduced Binary Logistic Regression (Firth, 1993), which uses penalized ML^22^. Cluster membership (TD or SLI) served as the dependent measure. Models with Firth’s correction were built using the *Brglm2* R package (Kosmidis, 2018). We included only a maximum of four background information variables as fixed factors in the model to avoid over-parametrization given the overall small sample size. Because regression analysis provides a way of adjusting for potentially confounding covariates included in the model, we entered the covariates into the model at once. To examine whether the diagnostic accuracy of the tasks is not compromised by language dominance and is only sensitive to risk factors for SLI, we built several regression models using Firth’s correction with PAM cluster membership as TD or SLI as the dependent variable. In each model, we entered LDI and the index of Positive_Early_Development (risk factors for SLI) in addition to two further background information variables reported to explain performance on LITMUS-SRT and NWRT (see Tuller et al., 2018) as covariates. The latter variables included AoO, LoE, SES. We also included chronological age as a covariate since working memory and cognitive capacities are rapidly growing in children and since language abilities of children tend to improve over time.

Background information variables were first scaled by the mean of their original variable to remove potential *non-essential* multi-collinearity between them (Dalal and Zickar, 2011) and to adjust the interpretation of the coefficients. Multi-collinearity among covariates was checked using the *Variance Inflation Factor (VIF)* after scaling, with a VIF value above 10 indicating serious multi-collinearity (Kutner et al., 2004). Correlations between the background variables are given in the Appendix.

## Results

### Overall Results on the German LITMUS NWRT and SRT

Kruskal-Wallis tests comparing performance scores of L1-dominant BiTDs to the other groups (MoTD, balanced-BiTD, L2-dominant-BiTD, MoSLI, and BiSLI) on NWRT_global, NWRT_LI, NWRT_LD, SRT_Id and SRT_Tar yielded significant results for all measures as shown in Table 5.

**Table 5:** The effect of (clinical) group membership on LITMUS-NWRT and SRT (Kruskal-Wallis tests) and pairwise comparisons (Mann-Whitney U tests).

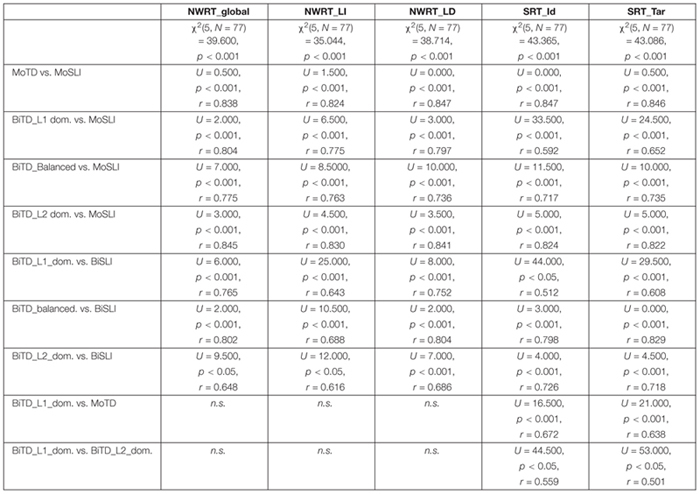

**FIGURE 2:**
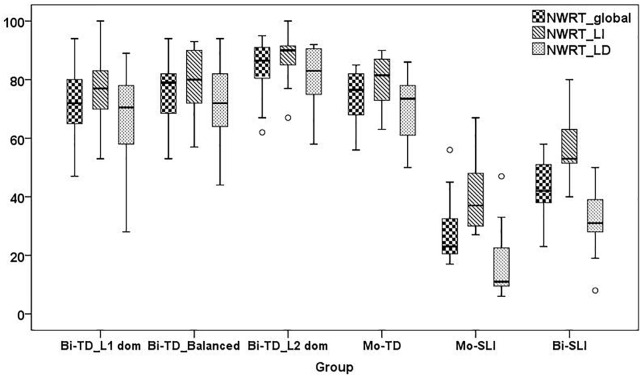
NWRT: % correct identical repetition (NWRT_global, NWRT_LI, and NWRT_LD).

**FIGURE 3:**
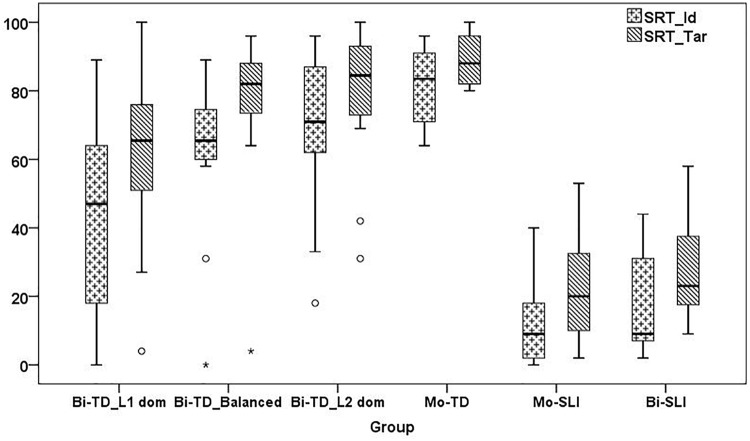
SRT: % identical repetition (SRT_Id) and correct production of target structure (SRT_Tar).

Subsequently, pairwise comparisons were carried out using Mann-Whitney U tests with Bonferroni-adjustment. Typically developing children performed significantly better than their language-impaired counterparts on all measures. All measures distinguish between MoTDs and MoSLIs as well as between BiTDs and BiSLIs regardless of language dominance: Moreover, all of the BiTD groups significantly outperformed MoSLIs. The comparisons yielded no significant differences between MoSLIs and BiSLIs on any of the aforementioned measures. Comparing MoTDs to the BiTDs split by dominance revealed no significant differences between MoTDs and balanced as well as L2-dominant BiTDs on either measure. Nevertheless, significant differences with large effect sizes were found between MoTDs and L1-dominant BiTDs as well as between L1-dominant and L2-dominant BiTDs for both SRT_Id and SRT_Tar but not for any of the NWRT measures **(see** Table 5). It should, however, be stressed that despite the observed significant differences in SRT_Id and SRT_Tar, L1-dominant BiTDs performed significantly better than MoSLIs and BiSLIs on both SRT measures. Figure 2 and 3 depict the overall performance of the groups in NWRT and SRT, respectively. An overview of significant pairwise comparisons is provided in Table 5.

In the next step, we collapsed all of the BiTDs into one group and ran partial correlation analysis controlling for age on their performance in SRT_Id and SRT_Tar and variables shown to influence performance on LITMUS-SRT including language dominance (see Tuller et al., 2018). Moderate positive correlations were found between LDI and performance on SRT_Id (*r* = 0.542, *p <* 0.001) and SRT_Tar (*r* = 0.586, *p <* 0.001), as well as SES and SRT_Id (*r* = 0.478, *p* = 0.001) and SRT_Tar (*r* = 0.431, *p* = 0.004). The analysis revealed a weak positive correlation between SRT_Id and LoE (*r* = 0.364, *p <* 0.05) and a weak negative correlation between SRT_Id and AoO (*r* = -0.348, *p <* 0.05), whereas the latter two correlations were not significant in case of SRT_Tar. Two multiple linear regression models were built for predicting performance of the BiTDs on SRT_Id and SRT_Tar. The following variables were entered into the model as independent variables: AoO, LoE, LDI, and SES. The results show that performance on SRT_Id in the BiTD group is predicted by LDI (β = 3.724, *T* = 2.922, *p* = 0.001), followed by LoE (β = 3.846, *T* = 2.287, *p* = 0.01), and SES (β = 3.424, *T* = 2.829, *p* = 0.001). However, for SRT_Tar only LDI and SES had significant effects in the full model: LDI (β = 4.480, *T* = 3.360, *p* = 0.001), SES (β = 2.914, *T* = 2.301, *p* = 0.01). The independent variables did not show multi-collinearity in the models (VIF < 3 for all independent variables).

Comparison of global performance of L1-dominant BiTDs to their monolingual, balanced, and L2-dominant peers as well as results of regression analyses show that language dominance was the first predictor to explain performance of the BiTDs on both SRT_Id and SRT_Tar, and point to the possibility that language dominance could compromise the diagnostic accuracy of the SRT if administered to bilinguals in their non-dominant language, here German. In order to examine this, we ran the k-medoid PAM-clustering to group the children into SLI vs. TD based on their performance scores on SRT and NWRT, determined the cut-off points between the clusters for each of the repetition measures and calculated the diagnostic accuracy for different combinations of sub-measures of the two. Next, regression analyses using Firth’s correction were carried out to examine whether language dominance contributed to results of PAM-clustering, i.e., assigning the children to the clinical vs. non-clinical cluster based on performance scores on the LITMUS repetition tasks. We examined this for SRT and NWRT separately as well as combined. LDI and Positive_Early_Development were entered as predictors for PAM cluster membership into all regression models in addition to two further background variables (age, AoO, LoE, SES).

**FIGURE 4:**
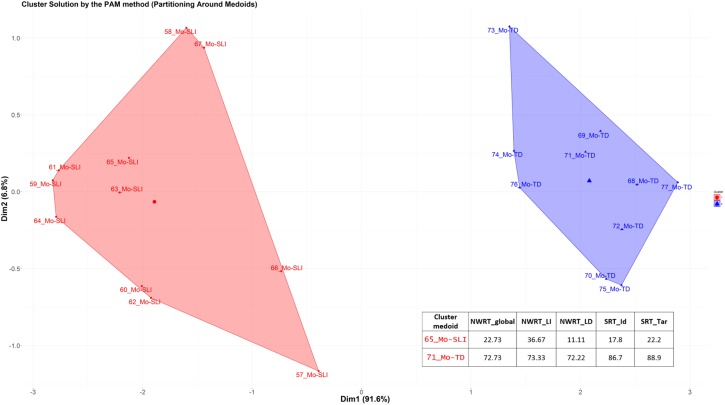
PAM-clustering of MoSLI+MoTD based on performance scores on SRT_Id, SRT_Tar, NWRT_global, NWRT_LI, and NWRT_LD.

Before applying the PAM clustering to our bilinguals, we first tested it on our monolingual data set. The following variables were entered in the cluster analysis simultaneously: NWRT_global, NWRT_LI, NWRT_LD, SRT_Id and SRT_Tar. The *Hopkins statistic* yielded a value of around 0.23 indicating clusterable non-random data, and the Gap Statistic revealed that the optimal cluster solution is 2. The clustering procedure resulted in a clear separation into two homogenous groups with two cluster medoids. The cut-off points (see section “Data Analysis”) separating the monolingual clinical cluster from the non-clinical one in our data sample were as follows: SRT_Id: 40%, SRT_Tar: 53.3% and NWRT_global: 45.45%, NWRT_LI: 60%, NWRT_LD: 47.22%. Figure 4 gives a visual representation of the k-medoid PAM-cluster analysis on monolingual data using the two-cluster solution. Cases belonging to the cluster on the right are identified as TD cases, while those in the cluster on the left as SLI cases. To facilitate computing sensitivity and specificity of the task, case numbers were combined with the clinical status as assigned by our classification procedure based on standardized assessment. As can be seen in Figure 4, all of the monolingual children assigned to the MoSLI group based on standardized test procedures belong to the clinical cluster, yielding a sensitivity of 100%, whereas all of the monolingual subjects classified as MoTD based on standardized test procedures belonged to the non-clinical cluster, which yields a specificity of 100%. We also ran the clustering procedure on SRT and NWRT separately, i.e., (SRT_Id+SRT_Tar) and (NWRT_global+NWRT_LI+NWRT_LD), respectively and obtained similar results. In a next step, we used regression analysis entering age^23^ as a single variable to check whether chronological age could explain cluster membership. Results of the latter analysis indicate that there is no association between age and the cluster variable (Firth: β = -0.03167, *Z* = -0.646, *p* = 0.519).

In order to check for overlap between BiTD and MoSLI, we applied the PAM-analysis to the MoSLI and BiTD children collapsed together using performance scores on both SRT and NWRT. The data yielded an H-value of around 0.18, which indicates clusterable non-random data with 2 as the optimal number of clusters. Before entering all five variables into the clustering procedure, we first carried out the clustering procedure based on performance on SRT_Id+SRT_Tar. Ctree models showed that SRT_Tar but not SRT_Id predicted cluster membership with a threshold of 53.3% separating the two clusters. All MoSLI children scored below cut-off and were thus assigned to the clinical cluster by the PAM algorithm, i.e., sensitivity = 100% with an LR+ = 6.29, whereas 37/44 BiTD children performed above threshold (specificity = 84.1%, LR- = 0.00). Age as a single variable in the regression model did not prove to be a predictor for cluster membership (Firth: β = -0.02849, *Z* = -1.238, *p* = 0.216). In the next step, we ran the PAM-analysis on NWRT_global, NWRT_LI and NWRT_LD. The clustering resulted in two clusters separated by a cut-off of 33.33% on NWRT_LD, which also is the primary predictor of clustering membership. 10/11 MoSLI children were assigned to the clinical cluster, yielding a sensitivity rate of 91% and 43/44 BiTD children performed above cut-off and were assigned to the non-clinical cluster giving a specificity of 98% with an LR+ = 39.56 and LR- = 0.092. Again, age was not a significant predictor for the cluster variable (Firth: β = -0.03618, *Z* = -1.293, *p* = 0.196).

**FIGURE 5:**
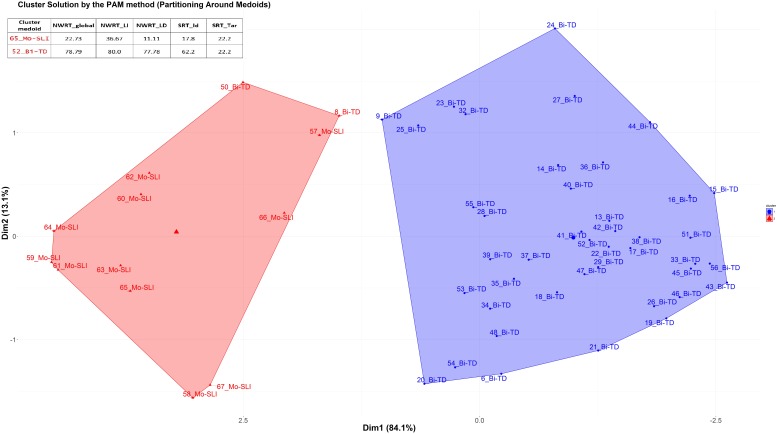
PAM-clustering of MoSLI+BiTD based on performance scores on SRT_Id, SRT_Tar, NWRT_global, NWRT_LI, and NWRT_LD.

Finally, both LITMUS-tasks were included in the PAM-analysis using the measures NWRT_global, NWRT_LI, NWRT_LD, SRT_Id and SRT_Tar. After entering all SRT and NWRT measures at once into the clustering procedure, 5 of the 7 BiTDs, who were assigned to the clinical cluster based on scores on SRT alone, changed membership from the clinical to the non-clinical cluster. A combination of both SRT and NWRT measures yielded 100% sensitivity (all MoSLIs belong to the clinical cluster) and 95% specificity (42/44 BiTDs belong to the non-clinical cluster) with an LR+ of 20 and an LR- of 0.00. An illustration of the result of the PAM cluster analysis on the MoSLI and BiTD data is given in Figure 5. Age at testing as a single variable did not play a significant role in predicting PAM cluster membership (Firth: β = -0.05145, *Z* = -1.824, *p* = 0.0681).

**FIGURE 6:**
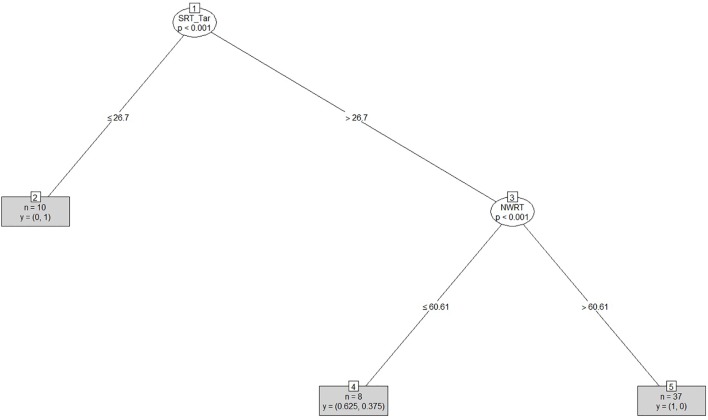
Conditional Inference Tree Analysis of the MoSLI+BiTD clustering result using SRT_Id, SRT_Tar, NWRT_global, NWRT_LI, and NWRT_LD as predictors.

A visual representation, a ctree, of the hierarchical structure of the most relevant linguistic variables for predicting PAM cluster membership illustrated in Figure 5 is provided in Figure 6. Within a ctree, only those variables serving as relevant to explaining the clustering results appear in the graph, where each relevant variable is represented by an oval circle and classification rules are represented by thresholds. Classification of cases starts at the top node (root). The second most important variable is one level below the top node. Classification then proceeds by moving down the branch until we arrive at a terminal node representing classification accuracy according to PAM clustering^24^, where classification accuracy is represented in squares (y). The two numbers next to “y” show the proportion of cases successfully classified and misclassified as SLI. The number of cases on that route is represented by “*n*.” Each classification route can be expressed in the form of if-then conditions with cut-offs. As can be seen in Figure 6, when all five measures are included in the clustering procedure, both SRT_Tar and NWRT_global are identified as significant contributors toward predicting PAM cluster membership. Classification of cases start at the top node occupied by SRT_Tar followed by the second most important variable “NWRT_global,” which is one level below the top node. Based on the hierarchical variable structure depicted in the ctree below, it becomes visible that 10 children whose scores on SRT_Tar were ≤26% were assigned to the clinical cluster. For subjects performing above 26% correct on SRT_Tar, performance on NWRT_global was taken into account giving rise to two roots: (a) if subject performs >26% on SRT_Tar and >60.61% on NWRT_global then assign to non-clinical cluster (TD), (b) if subject performs >26% on SRT_Tar but ≤60.61% on NWRT_global then assign to clinical cluster (SLI).

**FIGURE 7:**
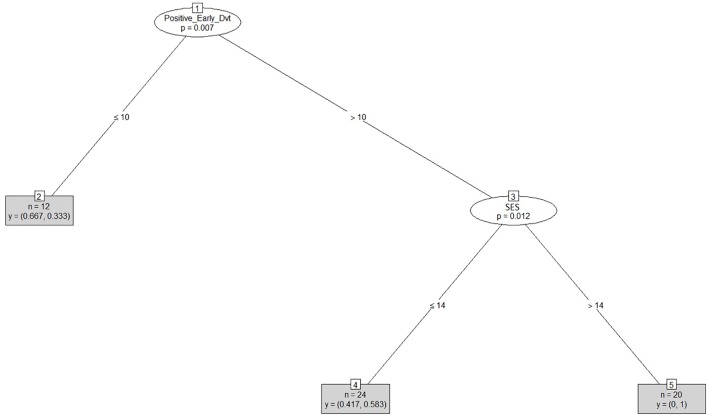
Conditional Inference Tree Analysis of the BiSLI+BiTD clustering result based on SRT_Id and SRT_Tar: hierarchical variable importance of background variables predicting cluster membership.

Turning now to results of bilingual children, we performed the PAM-analysis on all BiSLI and BiTD groups collapsed together based on the performance scores in the SRT and NWRT. The *Hopkins statistic* indicated regularly spaced data that are neither clustered nor random (H-value of around 0.2) and the Gap statistic suggested the two-cluster solution. Results of the PAM clustering based on performance of BiTDs and BiSLIs on SRT_Id and SRT_Tar were similar to those we obtained for BiTDs and MoSLIs (see Figure 5). 11/12 BiSLIs were assigned to the clinical cluster yielding a sensitivity of 91.7%, whereas 37/44 BiTDs were assigned to the non-clinical cluster giving a specificity of 84.1%, LR+ = 5.76, LR- = 0.10. The thresholds separating the bilingual clinical cluster from the non-clinical one were 33.3% for SRT_Id and 53.3% for SRT_Tar, whereby SRT_Tar was the main predictor for the clustering result (with the same cut-off of 53.3%). Regression analysis as well as ctree analysis showed that Positive_Early_Development (Firth: β = 1.0636, *Z* = 2.614, *p* = 0.001) followed by SES (β = 0.7843, *Z* = 2.033, *p* = 0.01) were significant predictors for cluster membership. Variables related to bilingualism, i.e., AoO, LoE and LDI, did not explain cluster membership. An illustration of hierarchical structure of variable importance with classification thresholds is depicted in Figure 7.

We ran the same clustering procedure on the bilinguals’ performance in NWRT using the variables NWRT_global, NWRT_LI and NWRT_LD. All BiSLI children were assigned to the clinical cluster yielding a 100% sensitivity; however, 9 BiTD children were assigned to the clinical cluster, i.e., only 35/44 BiTDs were assigned to the non-clinical cluster (specificity = 80%), LR+ = 5, LR- = 0.00. Ctree analysis showed that NWRT_global was the main variable predicting cluster membership with a cut-off 66.7%. Next, we ran Firth’s biased regression analysis on the clustering results for NWRT entering age, Positive_Early_Development, SES and LDI as fixed factors. Results showed that neither language dominance nor SES explained cluster membership based on NWRT_global. As expected, Positive_Early_Development was the main variable explaining the clustering result (Firth: β = 0.38996, *Z* = 2.626, *p* = 0.001). The other significant predictor for NWRT_global was chronological age (Firth: β = 0.05931, *Z* = 2.150, *p* = 0.01).

**FIGURE 8:**
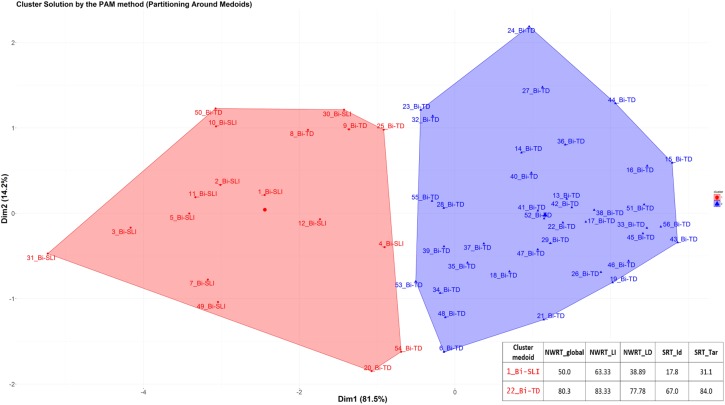
PAM-clustering of BiSLI+BiTD based on performance scores on SRT_Id, SRT_Tar, NWRT_global, NWRT_LI, and NWRT_LD.

Since NWRT_global is a composite score obtained by adding up scores of both of the language independent (NWRT_LI) and language dependent parts (NWRT_LD), we wanted to verify whether both of them were affected by the age factor. To achieve this, we ran the PAM-analysis on each of them separately. The results show that if clustering is solely based on performance on NWRT_LI upon a threshold of 73.3% (as established by ctree analysis), 10 BiTD children would be over-identified as having SLI, yielding a specificity of only 77%, LR+ = 4.385, LR- = 0.00. Both Positive_Early_Development (Firth: β = 0.38996, *Z* = 2.626, *p* = 0.01) and age (Firth: β = 0.05591, *Z* = 2.266, *p* = 0.01) were significant predictors for the clustering results (variables entered in the regression model: Positive_Early_Development, SES, LDI and age). Ctree analysis showed that the age threshold separating the two clusters based on NWRT_LI was 87 months (7;3 years). On the other hand, if the bilingual children in our data set are clustered based on performance in NWRT_LD alone, the diagnostic accuracy drastically improves: upon a 50% cut-off score, only 2/44 BiTD children are assigned to the clinical cluster, while all BiSLI children are classified as SLI, which yields 95% specificity and 100% sensitivity (LR+ = 20, LR- = 0.00). Positive_Early_Development was singled out as a predictor explaining cluster membership based on NWRT_LD (Firth: β = 0.30611, *Z* = 2.946, *p* = 0.001), i.e., the variables age, LDI and SES did not explain cluster membership. In the following step, we included all SRT and NWRT measures (SRT_Id, SRT_Tar, NWRT_global, NWRT_LI and NWRT_LD) in the clustering procedure. As can be seen in Figure 8, combing SRT with NWRT enhances the diagnostic accuracy: all of the BiSLI children (12/12) were assigned to the clinical cluster (100% sensitivity), while 39/45 BiTD children were assigned to the non-clinical cluster (87% specificity, LR+ = 7.692, LR- = 0.00).

**FIGURE 9:**
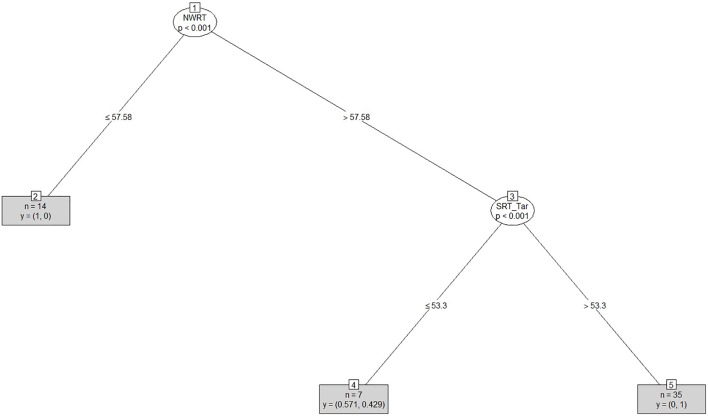
Conditional Inference Tree Analysis of the BiSLI+BiTD clustering result using SRT_Id, SRT_Tar, NWRT_global, NWRT_LI, and NWRT_LD as predictors.

Figure 9 shows that both NWRT_global and SRT_Tar were significant contributors toward predicting PAM cluster membership when all 5 variables are included in the clustering procedure. Classification of cases started at top node “NWRT_global” followed by the second relevant variable “SRT_Tar,” which is one level below the top node. According to the hierarchical variable structure illustrated in Figure 9, 14 children whose scores on NWRT_global were ≤57.58% were classified as SLI. In case of children with scores above 57.58% on NWRT_global, performance on SRT_Tar was taken into consideration leading to two roots: (a) if subject performs >57.58% on NWRT_global and >53.3% SRT_Tar, then assign subject to non-clinical cluster (TD), (b) if subject performs >57.58% on NWRT_global but ≤ 53.3% on SRT_Tar then assign to clinical cluster (SLI). Regression analysis using the previous four variables revealed that only Positive_Early_Development was a significant predictor for the clustering outcome (Firth: β = 0.39394, *Z* = 2.907, *p* = 0.001).

**FIGURE 10:**
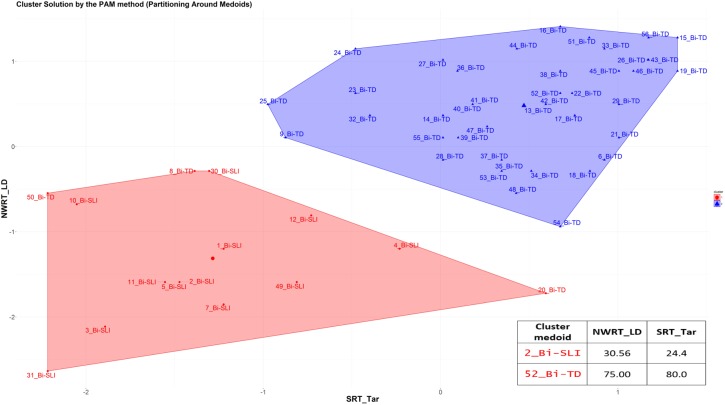
PAM-clustering of BiSLI+BiTD using performance scores on SRT_Tar and NWRT_LD.

To address research question (iii), we ran PAM clustering on bilingual data using scores of NWRT_LD and SRT_Tar in order to examine whether a combination thereof yielded the best diagnostic accuracy rates. Indeed, only 2/44 BiTD children were over-identified as having SLI (95% specificity) and all of the 12 BiSLI children were assigned to the clinical cluster (100% sensitivity) with an LR+ of 20 and an LR- of 0). The cut-off scores were 52.78% for NWRT_LD and 53.3% for SRT_Tar, with NWRT LD being the primary predictor for clustering results followed by SRT_Tar. Only Positive_Early_Development was a significant predictor of cluster membership (Firth: β = 0.39394, *Z* = 2.907, *p* = 0.001). The clustering results are depicted in Figure 10.

## Discussion and Conclusion

The purpose of this study was to evaluate the robustness of two LITMUS tools, German LITMUS-SRT and NWRT, against the influence of language dominance on their diagnostic accuracy for SLI in bilingual children. Since both tasks were designed to minimize bias against bilingual populations while being indicative of the presence of LI, we wanted to specifically verify whether the tasks were only sensitive to risk factors for SLI or whether background variables related to bilingualism, particularly, the degree of language dominance (as measured by relative amount of use and exposure to L1/L2) could influence the performance of BiTDs to an extent that would compromise their diagnostic accuracy. The second aim of the study was to investigate whether combining LITMUS-SRT (especially when scored by correct target structure) with NWRT yielded better diagnostic accuracy than single measures and helped avoid cases of misdiagnosis. Following our own research (e.g., Abed Ibrahim and Hamann, 2017; Hamann and Abed Ibrahim, 2017; Grimm and Hübner, in press), we particularly wanted to check whether a combination of German SRT_Tar and the language dependent part of the NWRT yielded higher diagnostic accuracy for identifying SLI in bilingual children than other combinations of measures. The former was found to be a fairer method than identical repetition for scoring SRT as it compensates for typical L2-errors such as lexical substitutions, while the latter was shown to maximize the performance gap between SLI and TD not only in monolinguals but also in bilinguals given its higher level of structural complexity.

In order to examine this, we first compared global performance of L1-dominant BiTDs to that of MoTDs, balanced and L2-dominant-BiTDs as well as to MoSLIs and BiSLIs. Results showed that although all three BiTD groups (regardless of their dominance) significantly outperformed MoSLIs and BiSLIs on all SRT and NWRT measures, L1-dominant-BiTDs were significantly outperformed by MoTDs and L2-dominant-BiTDs on both SRT_Id and SRT_Tar with large effect sizes (see Figure 2, 3). This echoes the findings of Meir (2018), who reported similar results for performance of Russian-Hebrew bilinguals on LITMUS-SRTs in their weaker heritage or societal language. Our results further showed that the performance gap between monolingual and bilingual SLI and TD groups was larger for NWRT_LD as opposed to the structurally less complex language independent part of the NWRT and the composite score of the two parts “NWRT_global” (see Figure 2). This is in line with previous work showing that the complexity factors involved in the NWRT_LD part (i.e., presence of trilateral onset clusters and /sC/ clusters violating the Sonority Sequencing Principle) is particularly challenging for language impaired children regardless of lingual status (Ferré et al., 2015; dos Santos and Ferré, 2018; Grimm and Hübner, in press).

Since language dominance was used as a categorical variable to classify BiTDs in our between-group comparisons, we had to entertain the possibility that the assumed dominance effect for L1-dominant children might have been caused by confounding variables such as age of onset of exposure to L2 (AoO), length of exposure to L2 (LoE) and SES. As for SRT_Id and SRT_Tar, we found moderate correlations between performance and language dominance as well as SES, in addition to weak correlations for SRT_Id with LoE and AoO. Regression analysis showed that language dominance was the key predictor explaining variance in the performance of the BiTDs on SRT_Id and SRT_Tar followed by SES. That AoO and LoE did not predict performance of BiTDs on the SRT was an expected outcome since the vast majority of the participants in our bilingual sample were either simultaneous or early successive and were exposed to German for at least 24 months at the time of assessment (see also Armon-Lotem, 2011 for similar results on L2-Hebrew-SRT).

The finding that language dominance influenced the performance of BiTDs on both measures of the LITMUS-SRT questioned its applicability for the identification of SLI in L1-dominant children when administered in their weaker language (German). To answer this, we used a prominent unsupervised machine learning technique, the Partitioning Around Medoids (PAM) for establishing an automatic classification of the monolingual and bilingual children in our data set as TD vs. SLI directly from their performance scores on SRT and NWRT without using information about their clinical status. Subsequently, we compared the participants’ clinical group membership revealed by PAM-clustering to their clinical status based on standardized assessment in L1/L2, and calculated sensitivity and specificity (diagnostic accuracy) levels of the tasks in isolation and combined. We also explored which combinations of the measures obtained from SRT_ Id, SRT_Tar, NWRT_global, NWRT_LI, and NWRT_LD yielded the highest diagnostic accuracy. Finally, we conducted regression analysis to investigate whether background variables other than risk factors for SLI, in particular language dominance (LDI), explained PAM-cluster membership as TD or SLI based on performance scores on SRT and/or NWRT. Since the index of Positive_Early_Development was shown to be a strong predictor for SLI in bilinguals (Boerma and Blom, 2017; Tuller et al., 2018), our premise was that if PAM-cluster membership can only be predicted by this index and not by language dominance or other background variables known to influence performance on repetition tasks (age, AoO, LoE, SES), then clustering of cases cuts across the SLI/TD dimension confirming that the LITMUS-SRT and NWRT are only sensitive to the presence of SLI and are not biased against bilingual children, who are non-dominant in the societal language.

In Hamann and Abed Ibrahim (2017), unsupervised (clustering) machine learning algorithms were only applied to the bilingual data, while Receiver Operating Characteristic curve (ROC) analysis was used to calculate sensitivity and specificity levels for the monolingual data. Given that ROC analysis uses “clinical status” (as assigned by standardized test procedures) as a dependent variable for predicting the sensitivity and specificity of a test, we wanted to verify this finding for the monolinguals using a method independent of “clinical status.” PAM-clustering solely based on scores in SRT_Id, SRT_Tar, NWRT_global, NWRT_LI, and NWRT_LD yielded even higher diagnostic accuracy than that in Hamann and Abed Ibrahim (2017). The fact that all of the subjects identified as MoSLI by standardized assessment belonged to the lower performing cluster, while all of the MoTDs belonged to the higher performing cluster (100% sensitivity and 100% specificity) provides additional evidence that these linguistically motivated tasks are very sensitive to the presence of LI in monolinguals and tap the core morphosyntactic and phonological deficits in SLI. The source of the improved diagnostic accuracy as compared to results based on ROC-analysis in Hamann and Abed Ibrahim (2017) is most likely the simultaneous inclusion of both tasks into the clustering procedure and the lower cut-off points obtained by applying ctrees to the PAM clustering. This is reminiscent of Armon-Lotem and Meir’s (2016) study, which reported an increase in diagnostic accuracy when LITMUS-SRT is supplemented by NWRT for Hebrew and Russian monolinguals. A further important result was that chronological age could not predict cluster membership for the age range in our monolingual data set.

After establishing that both LITMUS-SRT and NWRT were sensitive to SLI in monolinguals, we proceeded to address the frequently reported overlap between MoSLI and BiTD children (e.g., Håkansson and Nettelbladt, 1996; Armon-Lotem, 2010; Paradis, 2010; Hamann, 2012). PAM-clustering conducted on SRT scores entering both measures SRT_Id and SRT_Tar yielded good overall diagnostic accuracy (100% sensitivity and 84.1%^25^ specificity) with SRT_Tar being the leading variable for predicting cluster membership since it led to a better separation between the BiTD and MoSLI clusters. Several studies found this scoring method better suited for assessing morphosyntactic abilities in bilingual children, since it only focuses on the mastery of syntactic structure and does not penalize bilingual children for frequent L2-errors such as lexical substitutions (Armon-Lotem and Meir, 2016; Hamann and Abed Ibrahim, 2017; Hamann et al., 2017; Abed Ibrahim et al., 2018; Meir, 2018).

Next, we checked whether the overlap problem between MoSLI and BiTD could be overcome by using SRT in combination with NWRT. Indeed, including NWRT scores into the clustering procedure resulted in much better diagnostic accuracy with almost no overlap between MoSLI and BiTD (100% sensitivity and 95% specificity). As also reported in Armon-Lotem and Meir (2016), de Almeida et al. (2017), Boerma and Blom (2017), and Hamann and Abed Ibrahim (2017), the latter finding corroborates that a combination of LITMUS instruments assessing different areas of language ability helps to avoid cases of misdiagnosis. Among the five measures SRT_Id, SRT_Tar, NWRT_global, NWRT_LI and NWRT_LD, both SRT_Tar and NWRT_global were main predictors for clustering results with SRT_Tar being the more important contributor (see Figure 6). We further demonstrated that chronological age did not predict cluster membership here either.

As to the diagnosis of bilinguals, PAM clustering based on scores in SRT_Id and SRT_Tar resulted in good overall accuracy rates (91.7% sensitivity and 84.1% specificity). Interestingly, the same 7 BiTDs previously assigned to the clinical cluster upon comparison with MoSLIs were classified as SLI by the PAM as well showing that changing the reference group had no influence on the individual classification of the BiTDs. Again, SRT_Tar, which compensates for L2-errors, was the primary contributor toward the clustering results with a cut-off 53.3%, which is very close to the threshold obtained by *k*-means clustering in Hamann and Abed Ibrahim (2017). Of the five background variables considered for regression analysis, just two variables unrelated to bilingualism emerged as significant predictors for clustering membership: Positive_Early_Development followed by SES. The influence of language dominance, which was a significant predictor explaining the variance in the performance of the BiTDs in SRT_Id and SRT_Tar, was outweighed by the presence of risk factors for SLI and was rendered insignificant once the BiSLIs became part of the equation. This is consistent with the findings of Tuller et al. (2018), who found for the German children that Positive_Early_Development was the leading predictor for performance in SRT (followed by SES) over variables related to bilingualism.

The clustering solution based on NWRT_global, NWRT_LI and NWRT_LD scores yielded only fair diagnostic accuracy rates due to reduced specificity (specificity = 80%). NWRT_global emerged as the main predictor for clustering results. Regression analysis revealed that not only Positive_Early_Development (most important predictor) but also chronological age were significant predictors for clustering results based on performance scores in NWRT_global. Given that NWRT_global is a composite score computed by adding up performance scores in NWRT_LI, and NWRT_LD, and since Grimm and Hübner (in press) reported an overlap between MoSLI and BiTD on NWRT_LI and better discriminatory power for NWRT_LD in children aged 8;0 to 10;0 years, we ran cluster analyses on both subparts of the NWRT separately to check for age effects. The analysis revealed that in addition to Positive_Early_Development, cluster-membership based on NWRT_LI was predicted by chronological age with a threshold of 7;3 years, whereas cluster-membership based on NWRT_LD was not predicted by age and was only sensitive to risk factors for SLI. On the other hand, neither bilingualism related factors nor SES predicted cluster membership derived by performance scores on NWRT_global or subtests thereof. The latter result echoes what has been found for this type of NWRT in de Almeida et al. (2017) as well as in Tuller et al. (2018).

We have also shown that including all five SRT and NWRT measures in the clustering procedure enhances diagnostic accuracy for SLI in bilingual children, where NWRT_global and SRT_Tar were the main contributors explaining the results of the cluster solution. Interestingly, once SRT is combined with NWRT, only Positive_Early_Development emerges as a significant predictor for clustering results and SES does not play a role anymore, which is in line with the findings of Chiat and Polišenská (2016).

Given that clustering by scores on NWRT_LI appeared to be influenced by age, while NWRT_LD was only sensitive to risk factors of SLI (Positive_Early_Development) and since the SRT_Tar was the chief contributor toward clustering results when both SRT_Id and SRT_Tar were included in any clustering procedure on bilingual performance, we expected a combination of SRT_Tar and NWRT_LD to yield better diagnostic accuracy rates than other combinations of measures. Indeed, clustering based on performance scores on SRT_Tar and NWRT_LD yielded the highest diagnostic accuracy, where only Positive_Early_Development predicted clustering results. The crucial contribution of the structurally more complex NWRT_LD toward diagnostic accuracy is consistent with the robust effects of phonological complexity found in the respective studies (e.g., Gallon et al., 2007; Ferré et al., 2012), with clinical implications that phonological complexity can be used as a reliable indicator for SLI in both monolingual and bilingual children (see Grimm and Hübner, in press). Our results concerning the NWRT_LD part might seem at odds with results of other studies showing better diagnostic accuracy for *Crosslinguistic*-NWRTs over *Language-Specific*-NWRTs in bilingual populations, e.g., Boerma et al. (2015), Armon-Lotem and Meir (2016), and Boerma and Blom (2017). This can clearly be ascribed to differences in the construction of the tasks, which, as described in the section “The German LITMUS Nonword Repetition Task”, tap different aspects vulnerable in SLI (i.e., phonological working memory vs. phonological complexity), and differ considerably from each other, especially in their language dependent parts. Another possible reason for the poor diagnostic accuracy reported for the *Language-Specific*-NWRTs in the latter three studies might be relatively young age of their participants (5;0–6;0) compared to the age range in our sample (5;6–9;0), which covers the last year of preschool and the first 2–3 primary school years. A study by Rispens and Baker (2012) demonstrated that both lexical knowledge and discrimination ability significantly influenced performance on NWRT in 5-year-old MoTDs, while this kind of relation could not be attested for 8-year olds.

In line with our previous research, the results presented here and the fact that they emerge from unsupervised PAM-clustering clearly indicate that the German LITMUS- SRT and NWRT are promising tools for the identification of LI in bilingual populations with diverse dominance profiles. We replicated the finding that SRT_Tar is better suited than SRT_Id for the assessment of language abilities of bilingual children with German as L2 on a slightly larger group of children with a statistical method better suited for our data set. Even though dominance influences the performance of BiTDs, especially in the SRT, we demonstrated that the diagnostic accuracy of these tools is not compromised by language dominance: while risk factors for SLI were significant predictors for clinical status in all models, language dominance did not contribute at all to explaining results of any of the clustering procedures. Moreover, our results confirmed that using a combination of tasks, each emphasizing a different aspect of language ability, enhances diagnostic accuracy and helps avoid cases of misdiagnosis. As a last promising result, we showed that using SRT_Tar in conjunction with NWRT_LD renders the best diagnostic accuracy so far obtained in studies on similarly constructed tasks, where the combination of measures is only sensitive to risk factors for SLI, but not to language dominance nor to SES, which is not achieved by many tasks. We therefore feel confident in pursuing these investigations in order to be able to provide useful and easy to administer L2-tools for clinical use in bilingual contexts. Finally, it should be noted that vast majority of the bilingual children in our sample were either simultaneous or early successive bilinguals, who had at least 2 years of exposure to the L2. Thus, future research should focus on testing the applicability of this particular combination of tasks to bilinguals with less exposure to the L2.

## Author Contributions

LAI developed the theoretical framework, gathered and evaluated the data. IF conducted the statistical analysis. All authors wrote the manuscript.

## Conflict of Interest Statement

The authors declare that the research was conducted in the absence of any commercial or financial relationships that could be construed as a potential conflict of interest. The reviewer AG and handling Editor declared their shared affiliation.

## Footnotes

1 We are aware of the recent consensus on using the term “Developmental Language Disorder-DLD” for unexplained language impairment in the absence of primary deficits. Nevertheless, we chose to refer to this disorder as SLI in this paper for the sake of continuity with much of the existing literature on bilingual SLI and our own collaborative research within the Franco-German project “BiLaD.”

2 Linguistic operations such as recursion and hierarchical non-local dependencies between grammatical elements (van der Lely, 2005, p. 53).

3 Here, language-dependency is viewed as “an abstract phonological property rather than a lexical or sub-lexical property” (Grimm and Hübner, in press).

4 Input and output are collapsed together (interaction from interlocutor to child and from child to interlocutor).

5 Five additional points were allotted to number of years in elementary school as part of the exposure indices in France, but not in Germany, where children join elementary school between the age of 6 and 8.

6 Total length of exposure (LoE) is calculated by subtracting age of onset of systematic sustained exposure to the respective language from the chronological age.

7 Contrary to Unsworth (2016) and Unsworth et al. (2018), PaBiQ’s language use estimates were not only limited to the “inside home context” but also cover the “outside home context.”

8 A number of studies such as Balladares et al. (2016) showed that the influence of SES on repetition tasks diminishes after controlling for vocabulary sizes indicating that the effect of SES is primarily ascribed to smaller vocabulary sizes in children with lower SES.

9 Intervention can be defined syntactically as in Rizzi (2004, 2013) modeled on work by Gibson (1998) or Gordon et al. (2001).

10 German is a verb-second (V2) language, which requires the finite verb (marked for tense and agreement) to be the second constituent of main (root) clauses. Like other Germanic languages (except for English), it allows different types of constituents (e.g., complements, adverbials) to occupy sentence initial position in main clauses (e.g., topicalization), where only one constituent can linearly precede the finite verb (cf. Chomsky, 1986; Eisenberg, 1999).

11 Case errors are not disregarded if they are crucial for the realization of the targeted structure, e. g., object relatives and topicalized sentences.

12 The narrative samples of the children were evaluated by linguistically trained native speakers (L1 and L2) according to certain markers: e.g., subject-verb-agreement and sentential complexity, i.e., presence of embeddings.

13 Following the recommendation of Thordardottir (2012, 2015), the monolingual -1.25 SD cut-off criterion used by Tomblin et al. (1996) was adapted according to the dominance status of the language being assessed. Accordingly, we used a criterion of -1,5 SD if the child was evaluated in her dominant language, and a cut-off of -2,25 SD if the child’s weaker language was being assessed. In case of balanced bilinguals, the cut-off criterion was set at -1,75 SD for both languages.

14 In case of children older than the norming sample, we consulted with the test authors concerning the possibility of norm-extension, e.g., LiSe-DaZ and ELO-L.

15 Norms are only available for the Lebanese version. Due to linguistic proximity between Lebanese, Syrian, Jordanian and Palestinian Arabic, the Lebanese norms can be applied to the latter varieties with the caveat that the socio-cultural context may differ. In case of Maghreb (Moroccan, Tunisian, and Algerian) dialects, norms should be viewed with caution, especially in borderline cases.

16 Two of them were cases of selective impairment in the L2, i.e., they performed below cut-off in the L1, and only showed deficits in morphosyntax in the L2 (grammatical SLI) plus SLI markers in the speech samples in both languages. The other two were L2-dominant and performed below-cut-off in all of the domains in the L2, but slightly above cut-off in the L1. Since SLI markers were present in their speech samples, we classified them as BiSLI

17 This reflects the advice frequently given to parents of bilingual children with atypical language development that they should restrict parent-child interactions to the societal language to avoid aggravating the existing language difficulties, which in turn means less exposure to the L1.

18 Note that language dominance was used as a categorical variable in this step.

19 The notion of similarity in the clustering approach is operationalized as Euclidean distance.

20 Following Plante and Vance (1994), good diagnostic accuracy is given when specificity and/or specificity rates are ≥90%. Rates between 80 and 90% are viewed as fair.

21 LR+ values ≥10 are highly indicative of the presence of language impairment, LR- values ≤0.10 highly indicate the absence of an impairment, LR+ values ≥3.0 and LR- values ≤0.3 are considered to be clinically suggestive, while LR+ values <3.0 and LR- values >0.3 are viewed as clinically uninformative (cf. Dollaghan and Horner, 2011).

22 We used Firth’s Bias-Reduced Logistic Regression to step around the following statistical concerns. Given our small sample, Long (1997) advises against maximum likelihood estimation in logistic regression with less than 100 cases. A second confound is that in small samples maximum likelihood estimates in binary logistic regression models are not powerful because there can be a complete- or quasi-complete separation along one covariate (Rainey, 2016). That is, for a given combination of covariates the outcome can be predicted perfectly. To avoid separation, Peduzzi et al. (1996) suggest that the number of positive outcome events (i.e., the smaller number of binary outcomes) divided by the number of independent variables should be more than 10.

23 SES information is only available for the bilingual participants.

24 The classification accuracy given in squares within the ctree refers only to that of PAM clustering and does not represent diagnostic accuracy of cluster membership as measured by comparing cases identified as SLI or TD by the PAM to the clinical status assigned based on our classification procedure outlined in section “Participants.”

25 It is worthwhile mentioning that three of the 7 BiTD children assigned to the clinical cluster started acquiring L2 German after the age of five and spent their first years in an exclusive L1-environment. The latter children demonstrated high rates of determiner errors, especially case and gender errors which could affect the realization of target structure of a sizable proportion of the test items of the LITMUS-SRT (see also Abed Ibrahim et al., 2018).

## APPENDIX

**Table A1:** Correlations between background variables for BiTD and BiSLI groups collapsed.

			Age	Positive_Earl_Dvt	AcO	SES	LDI	LoE
Spearman-Rhc	Age	Corr. coeff.	1.000	0.032	0.207	0.237	0.086	0.445^∗∗^
		sig. (2-tailed)		0.816	0.126	0.078	0.514	0.001
		*N*	56	56	56	56	56	56
	Positive_Early_Dvt	Corr. coeff.	0.032	1.000	0.103	0.064	-0.057	-0.146
		sig. (2-tailed)	0.816		0.450	0.639	0.676	0.282
		*N*	56	56	56	56	56	56
	AoO	Corr. coeff.	0.207	0.103	1.000	-0.051	-0.351^∗∗^	-0.670^∗∗∗^
		sig. (2-tailed)	0.126	0.450		0.711	0.008	0.000
		*N*	56	56	56	56	56	56
	SES	Corr. coeff.	0.237	0.064	-0.051	1.000	0.269^∗^	0.231
		sig. (2-tailed)	0.078	0.638	0.711		0.045	0.087
		*N*	56	56	56	56	56	56
	LDI	Corr. coeff.	0.089	-0.057	-0.351^∗∗^	0.269^∗^	1,000	0.428^∗∗^
		sig. (2-tailed)	0.514	0.679	0.008	0.045		0.001
		*N*	56	56	56	56	56	56
	LoE	Corr. coeff.	0.44^∗∗^	-0.146	-0.670^∗∗∗^	0.231	0.428^∗∗^	1.000
		sig. (2-tailed]	0.001	0.282	0.000	0.087	0.001	
		*N*	56	56	56	56	56	56

*^∗^The correlation is significant at the level of 0.05 (2-tailed).*

*^∗∗^The correlation is significant at the level of 0.01 (2-tailed).*

*^∗∗∗^The correlation is significant at the level of 0.001 (2-tailed).*
